# Microglial and peripheral immune priming is partially sexually dimorphic in adolescent mouse offspring exposed to maternal high-fat diet

**DOI:** 10.1186/s12974-020-01914-1

**Published:** 2020-09-05

**Authors:** Maude Bordeleau, Chloé Lacabanne, Lourdes Fernández de Cossío, Nathalie Vernoux, Julie C. Savage, Fernando González-Ibáñez, Marie-Ève Tremblay

**Affiliations:** 1 Integrated Program in Neuroscience, McGill University, Montreal, QC Canada; 2 Axe neurosciences, Centre de recherche du CHU de Québec–Université Laval, Québec, QC Canada; 3 Cerebral Imaging Center, Douglas Mental Health University Institute, McGill University, Montréal, QC Canada; 4 Department of Neurosciences, University of California, La Jolla, San Diego, CA USA; 5 Département de médecine moléculaire, Université Laval, Québec, QC Canada; 6 Department of Neurology and Neurosurgery, McGill University, Montréal, QC Canada; 7 Division of Medical Sciences, University of Victoria, Victoria, BC Canada; 8 Department of Biochemistry and Molecular Biology, University of British Columbia, Vancouver, BC Canada

**Keywords:** Hippocampus, Immune priming, Maternal high-fat diet, Microglia, Sex difference

## Abstract

**Background:**

Maternal nutrition is critical for proper fetal development. While increased nutrient intake is essential during pregnancy, an excessive consumption of certain nutrients, like fat, can lead to long-lasting detrimental consequences on the offspring. Animal work investigating the consequences of maternal high-fat diet (mHFD) revealed in the offspring a maternal immune activation (MIA) phenotype associated with increased inflammatory signals. This inflammation was proposed as one of the mechanisms causing neuronal circuit dysfunction, notably in the hippocampus, by altering the brain-resident macrophages—microglia. However, the understanding of mechanisms linking inflammation and microglial activities to pathological brain development remains limited. We hypothesized that mHFD-induced inflammation could prime microglia by altering their specific gene expression signature, population density, and/or functions.

**Methods:**

We used an integrative approach combining molecular (i.e., multiplex-ELISA, rt-qPCR) and cellular (i.e., histochemistry, electron microscopy) techniques to investigate the effects of mHFD (saturated and unsaturated fats) vs control diet on inflammatory priming, as well as microglial transcriptomic signature, density, distribution, morphology, and ultrastructure in mice. These analyses were performed on the mothers and/or their adolescent offspring at postnatal day 30.

**Results:**

Our study revealed that mHFD results in MIA defined by increased circulating levels of interleukin (IL)-6 in the mothers. This phenotype was associated with an exacerbated inflammatory response to peripheral lipopolysaccharide in mHFD-exposed offspring of both sexes. Microglial morphology was also altered, and there were increased microglial interactions with astrocytes in the hippocampus CA1 of mHFD-exposed male offspring, as well as decreased microglia-associated extracellular space pockets in the same region of mHFD-exposed offspring of the two sexes. A decreased mRNA expression of the inflammatory-regulating cytokine *Tgfb1* and microglial receptors *Tmem119*, *Trem2*, and *Cx3cr1* was additionally measured in the hippocampus of mHFD-exposed offspring, especially in males*.*

**Conclusions:**

Here, we described how dietary habits during pregnancy and nurturing, particularly the consumption of an enriched fat diet, can influence peripheral immune priming in the offspring. We also found that microglia are affected in terms of gene expression signature, morphology, and interactions with the hippocampal parenchyma, in a partially sexually dimorphic manner, which may contribute to the adverse neurodevelopmental outcomes on the offspring.

## Background

Maternal obesity and dietary overconsumption are risk factors for several health conditions in the offspring, from metabolic syndrome to neurodevelopmental disorders [1–3]. In fact, excess weight has been on the rise in both middle and high income countries, affecting over one third of the global population and about 38.9 million pregnant women worldwide [4]. This increase in the number of overweight or obese pregnant women has been linked to several elements of modern-day environment such as urbanization rate and increased caloric supply [4], which are often associated with increased fast food consumption [5]. This global issue stresses the importance of studying the impact of energy-dense, high-sugar, and high-fat food diets during pregnancy [5].

Providing high-fat diet (HFD) to animal models mimics the excessive intake of energy-dense, high-sugar, and high-fat food in human [6]. Of these overconsumed nutrients, fat is of utmost importance for brain growth and development [7]. However, several independent studies using different animal models of maternal (m)HFD have shown a broad range of lasting behavioral alterations in the offspring related to neurodevelopmental disorders from increased anxiety-like behaviors [8–13] to cognitive [8, 14–18], social, and motor deficits [9]. Although the mechanisms linking mHFD to the neurodevelopmental alterations remain unclear, several pathological processes such as decreased placental function, hormonal dysregulation, epigenetic alterations [2], and increased central as well as systemic inflammation [1] have been proposed.

In the past decade, epidemiological studies identified “maternal immune activation” (MIA), which refers to maternal systemic inflammation, as a risk factor for several neurodevelopmental disorders [19–22]. This has mostly been observed and researched in the context of infection, where a tight association between bacterial or viral (e.g., influenza) infections during pregnancy and a higher incidence of neurodevelopmental disorders, such as schizophrenia, was uncovered in the progeny [19, 20, 23]. It is now known that MIA is not limited to infection [19, 20, 23] and may occur after exposure to a large variety of maternal environmental risk factors common to modern-day life including stress [24, 25], smoking, alcohol consumption [26], air pollution [27, 28], and dietary imbalance or overconsumption such as HFD [1]. Postmortem studies revealed neuroimmune alterations in the brain of individuals with developmental disorders, as highlighted by the alteration of microglia—the brain-resident macrophages, in terms of morphology and gene expression signature [29–31], together with changes in the brain levels of cytokines—the molecules involved in signalling and modulation of the inflammatory state, among other important functions [19, 20, 23].

In animal models of mHFD, the inflammatory status in the offspring brain, notably in hippocampus, has been characterized by measuring changes of several inflammatory mediators during the lifespan. The mRNA expressions of *Cd11b* and *Tlr4*—two genes involved in the innate immune response—were found to be increased in the hippocampus of male and female rat offspring as early as postnatal day (PND)1, followed by *interleukin* (*Il*)*1β* at juvenile stages [8]. During adolescence, both male and female rat offspring exhibited greater levels of the cytokine *Il6*, as well as of the inflammatory regulators *nuclear factor κb* (*Nfκb*) and *mitogen-activated kinase protein* (*Mkp*) *1* in the hippocampus [32]. Adolescent male rat offspring also expressed increased levels of *Nfκb inhibitor* (*IκB*) in the hippocampus. Later in adult life, a few studies observed no change in cytokines [10] while others found greater expression of *Il1β* in the hippocampus of male rats [8], and lower expression of *IκB* and *Il1ra* in the hippocampus of female rats [13] exposed to mHFD. However, it remains unclear if this increased gene expression of inflammatory mediators is directly linked to a global inflammatory status or to altered microglial activities in the offspring brain. So far, the reported microglial changes induced by mHFD in the offspring brain comprise a higher density of ionized calcium-binding adapter 1 (IBA1)-positive (^+^) cells in the hippocampus of adult male and female rats [8] suggesting changes in microglial population or recruitment of peripheral macrophages to the brain.

Studies performed in animal models and human postmortem samples have revealed that microglia modulate key neurodevelopmental processes—cell migration and maturation, followed by the formation of neuronal circuits and myelination—in a sexually dimorphic manner [33–35]. Microglia also contribute to neuronal circuit refinement during adolescence [36] and adulthood [29, 35, 37–39]. Paralleling the immune alterations described above, mHFD was shown to be associated with synaptic changes resulting in decreased synapses density and/or spines density and stability in rodent offspring across brain regions and stages of the lifespan [16, 25, 40–42] including the hippocampus of adolescent mice [16]. Microglial cell density, distribution, gene expression signature, physiological functions, and/or response to immune challenges may be altered upon exposure to various environmental factors, thus impacting on their sculpting of the brain network [43, 44]. This phenomenon is referred to as immune priming [43–45]. Studying microglial priming during sensitive periods like adolescence, in which the brain and especially the hippocampus experience important synaptic changes, may help to understand the pathological cascade underlying mHFD.

Using a mouse model, we explored the changes of microglia in the offspring exposed to mHFD (enriched in both saturated and unsaturated fats) vs control diet (CD) [8]. Specifically, we investigated microglia-related gene expression in whole hippocampus, as well as microglial density, distribution, morphology, and ultrastructure in the dorsal hippocampus CA1. We focused on this region considering its crucial importance for memory, learning [46, 47], and executive [48] functions that are affected with MIA [19–22] including with mHFD [8, 15, 16, 42]. The measurements were made at PND30, which corresponds to early adolescence [49]. To characterize our model, we also assessed metabolic changes in the mothers and offspring, together with peripheral immune priming in the adolescent offspring, using a single systemic immune challenge with lipopolysaccharide (LPS) at PND30. Male and female offspring were compared in all experiments to investigate possible sex differences.

## Methods

### Animal and tissue processing

All animal protocols were approved by McGill University’s Facility Animal Care Committee under the guidelines of the Canadian Council on Animal Care. Mice were submitted to 12 h dark/light cycles (8:00–20:00) with an ad libitum access to water and food. A total of 116 females were used, and 50 females got pregnant after mating, out of which 16 lost their litter (e.g., spontaneous abortion in late pregnancy, cannibalism, leaked water bottle). A total of 112 offspring was used to study the effects of mHFD.

**Fig. 1:**
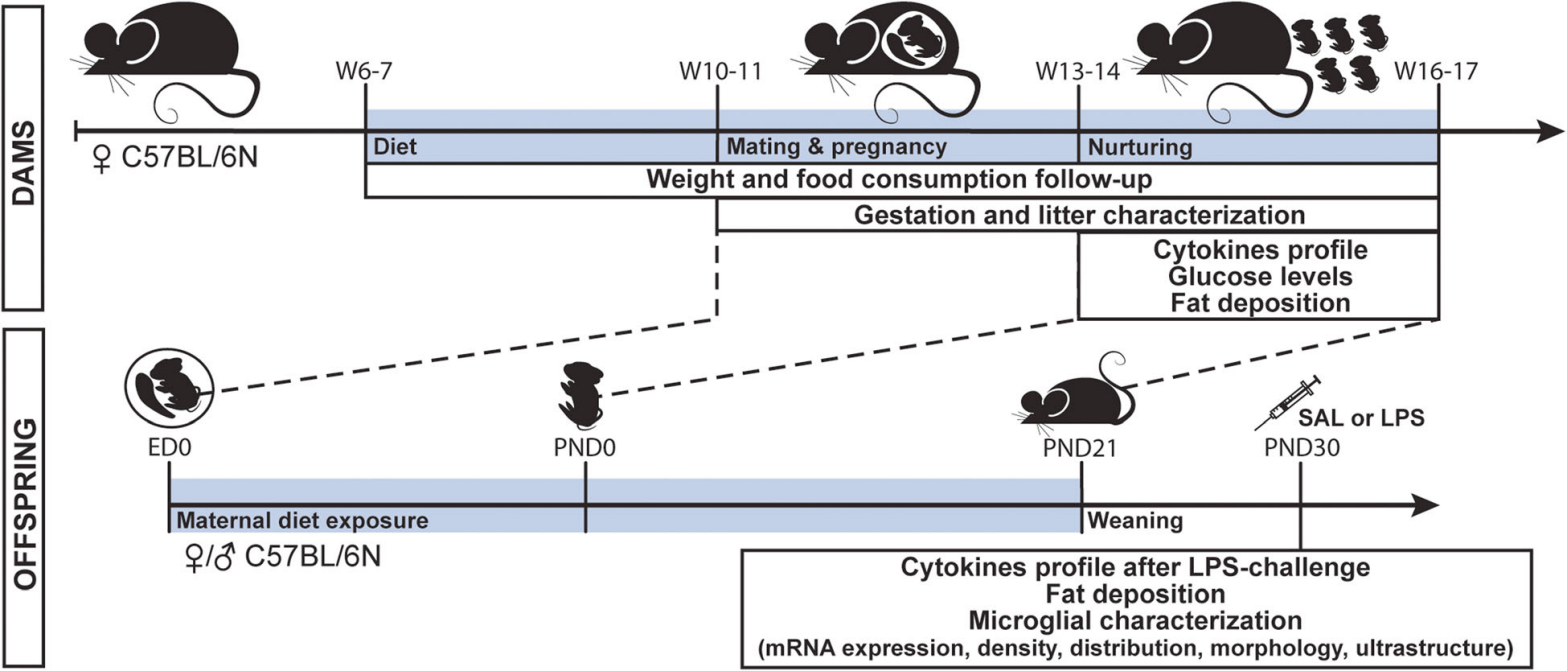
Experimental outline of HFD protocol on the dams and the offspring. Diet protocol period is identified by a pale blue-colored bar on the dams and the offspring timelines. At PND21, all offspring were on normal chow diet before sacrifice at PND30. Part of the animals received an intraperitoneal LPS injection 8 h before characterizing their peripheral inflammatory profile. ♀, female; ♂, male; E, embryonic days; LPS, lipopolysaccharide; P, postnatal days; SAL, saline; W, weeks

Paired-housed C57BL/6 N female mice aged 5–6 weeks were obtained from Charles River (St-Constant, QC, Canada) and habituated for 1 week prior to the protocol onset. They were then provided with either a HFD (diet rich in saturated [36% of total fat] and unsaturated fats [monounsaturated 41% of total fat and polyunsaturated 23% of total fat], 60.3% calories from lipids [3.075 kcal/g], 18.3% calories from proteins [0.933 kcal/g], and 21.4% calories from carbohydrates [1.091 kcal/g]; Teklad TD.06414, ENVIGO, Indianapolis, IN, USA; Supplementary Figure 1 e) or CD (13% calories from lipids [0.377 kcal/g] composed of saturated [17.6% of total fat] and unsaturated fats [monounsaturated 20.6% of total fat and polyunsaturated 61.8% of total fat], 20% calories from proteins [0.580 kcal/g], and 67% calories from carbohydrates [1.943 kcal/g]; Teklad 2014, ENVIGO; Supplementary Figure 1e) ad libitum starting 4 weeks prior to mating, throughout gestation and nurturing, until weaning of their litter (Fig. 1). After mating, pregnant female were housed alone, then with their litter. During the diet protocol, food consumption and weight were recorded twice per week in the dams to identify potential metabolic changes. Food consumption was averaged by number of animals housed and consumed food. About 4 h after their active phase with food ad libitum (between 12:00 and 13:00), blood samples were collected by submandibular puncture after 8 weeks on the diet to evaluate the inflammatory profile and assess circulating glucose levels. No fasting was performed since at that timepoint dams were also used either for cytokines assessment or fat distribution, and we did not want fasting to interact with these measures. Dams were anesthetized with a rodent cocktail (0.3 mL/100 g) containing ketamine [100 mg/mL], xylazine [20 mg/mL], and aceprozamine [10 mg/mL], fresh-decapitated at the end of the protocol, after 9–10 weeks on the diet, and their fat deposits were dissected (Fig. 1).

Male and female offspring were compared to study mHFD effects in the two sexes. During the study, animals presenting major anomalies (e.g., unopened or abnormal eye, dwarf or severe tooth malformation) were excluded from the experimental groups and sacrificed at weaning. Only 1–2 offspring of each sex per litter were used for each experiment to prevent litter effects. At PND21, the offspring were weaned and switched to CD. Offspring from same litter were housed together (2 to 5 animals due to animal housing restriction and birth-timed limitations) with sufficient resources (food, water, nesting) to prevent any competitive behavior. Of note, offspring’s weight gain between weaning and PND30 was similar regardless of the housing number. At PND30, offspring were anesthetized with rodent cocktail, and their blood, brain, and fat tissues were collected. One cohort of offspring (*n* = 5–6 animal/sex/diet) was fresh-decapitated, their brain rapidly extracted, and the hippocampus dissected, flash-frozen on dry ice, and stored at − 80 °C until mRNA analysis by real-time quantitative polymerase chain reaction (rt-qPCR). Another cohort of offspring was anesthetised as described above, perfused with 15 mL of phosphate-buffered saline (PBS) followed by ~ 180 mL of 4% paraformaldehyde (PFA) in [50 mM] phosphate-buffer (PB) for histological analysis (*n* = 5 animals/sex/diet). PFA-fixed brains were post-fixed in 4% PFA for 24 h at 4 °C, then immersed in 30% glucose solution (in [50 mM] PBS), pH = 7.4) at − 4 °C for 48 h and flash-frozen. Frozen brains were cut into 30 μm coronal sections using a cryostat (CM3050S, Leica Biosystems, Wetzlar, Germany) and stored free-floating in cryoprotectant (30% ethylene glycol, 30% glycerol in [50 mM] PBS, pH = 7.4) at − 20 °C until use for histochemistry. The last cohort of animals was anesthetised as described above, flushed with 15 mL of PBS, and perfused with 75 mL of 3.5% acrolein in [100 mM] PB (pH = 7.4) and 150 mL of 4% PFA in [100 mM] PB (pH = 7.4) for ultrastructural analysis (*n* = 4 animals/sex/diet). PFA/acrolein-fixed brains were post-fixed for 2 h in 4% PFA at 4 °C, washed in PBS, cut into 50 μm coronal sections with a vibratome (VT1200S, Leica Biosystems), and stored in cryoprotectant solution at − 20 °C until use (Fig. 1).

#### Adolescent peripheral immune challenge

To evaluate peripheral immune priming, a subset of animals (*n* = 5–7 animals/sex/diet/treatment) received a single intraperitoneal injection at PND30, of either 100 μg/kg lipopolysaccharide (LPS) from *Escherichia coli* O111:B4 (cat# 62325, Millipore Sigma, Burlington, MA, USA)—a component of the gram-negative bacterial wall used to model an inflammatory response—or saline (SAL; 0.9% NaCl solution, cat# 01966010, Hospira, USA). Eight hours after the administration, animals were anesthetized with rodent cocktail (0.3 mL/100 g) and decapitated. This timepoint corresponds to the resolution of inflammation after the peak usually seen ~ 2–4 h in the periphery and ~ 4–6 h in adult mouse brain [50–53]. Trunk blood was collected in heparinized tubes and centrifuged at 3600 rpm for 10 min. Plasma was collected and flash-frozen and stored at − 80 °C until analysis by multiplex-ELISA (see section “Cytokines measurement by multiplex-ELISA”) (Fig. 1).

### Physiological changes

To determine the phenotype induced by our diet model, a thorough characterization of the gestation and litter, as well as metabolic changes including weight, food intake, fat distribution, and glucose levels was performed in the dams. The methods and results are presented as Supplementary material.

### Cytokines measurement by multiplex-ELISA

To determine changes in the inflammatory profile of the dams, peripheral inflammation was assessed at the end of nurturing (after 8–9 weeks on the diet) (*n* = 5–6 dams/diet). Similarly, the peripheral inflammatory profile of the pubertal offspring (PND30) was characterized in homeostatic (SAL-treated) vs immune (LPS-treated) contexts (*n* = 5–6 animals/sex/diet/treatment). Levels of the cytokines IL-1β, IL-6, IL-10, IL-17, and tumor necrosis factor (TNF)-α were measured in maternal and offspring plasma using Luminex Multiplex Assay (MILLIPLEX MAP kit Mouse Cytokine/Chemokine Magnetic Bead Panel, cat# MCYTOMAG-70 K, Millipore Sigma). Plasma samples were diluted 1:2 by combining 30 μL of plasma with the provided drive fluid buffer. Immunoassays were performed following the manufacturer’s instructions. Diluted plasma was incubated overnight (~ 16 h) with primary antibodies at 4 °C, 1 h with detection antibodies at room temperature, and 30 min with Streptavidin-beads at room temperature. Samples were resuspended in 150 μL drive fluid and run through the Luminex MAGPIX to assess cytokines levels with the xPONENT software (v4.2.1324.0). Acquisition was done using manufacturer’s recommendation for the volume-uptake of sample (100 μL), the gate setting (8000–15,000), and the reporter gain (default, low PMT).

### Real-time quantitative polymerase chain-reaction

At PND30, the two hippocampi of each offspring (*n* = 5–6 animals/sex/diet) were homogenized in Trizol (cat#15596-026, Ambion, Austin, TX, USA), and RNA was extracted using the Trizol/chloroform method followed by an isopropanol precipitation. The RNA pellet was washed once in 75% ethanol, let dry, then eluted in Nuclease-free water (cat#AM9937, Ambion). Samples were dosed using the NanoDrop ND-1000 kit (ThermoFisher Scientific).

Genomic DNA was removed from 1 μg isolated RNA sample by enzymatic degradation (cat# G488, Applied Biological Materials Inc, Richmond, BC, Canada). Purified RNA was used to obtain complementary DNA (cDNA) by reverse transcriptase reaction with iScript 5× MasterMix (cat#1708890, BioRad Laboratories, Hercules, CA, USA) using a TI thermocycler (Biometra, Göttingen, Germany). Using optimal dilution of cDNA, rt-qPCR was performed with the SybrGreen technology using a LightCycler 480 II (Roche, Basel, Switzerland). The LightCycler 480 Software (v.1.5.1.62; Roche) automatically determined cycle threshold (Ct) as the linear portion of the amplification, as suggested per manufacturer’s guidelines. After each rt-qPCR, melting curve analysis was used to confirm proper amplification of a unique target.

Rt-qPCR was used to measure downstream inflammatory mediators *Nfκb* and *Cox2* (after 8 h LPS-induced immune challenge), microglial function-related genes *Tmem119*, *Aif1*, *Trem2*, *Cx3cr1*, homeostatic-regulating factor *Tgfb1* as well as housekeeping genes *Rpl32* and *Gapdh* (primers detailed in Table 1). For each gene’s primer pairs, dilution standard curve with a pool sample (1:1, 1:5, 1:25, 1:125, 1:625, 1:3125; 1:15,625) was used to determine optimal dilution cDNA (from 1 μg RNA reverse transcriptase reaction), and melt curve analysis was used to confirm specificity of the primer pairs. *Rpl32* obtained more stable Ct (intersample, 0.33%CV; intrasample, 0.29%CV) compared to *Gapdh* (intersample, 3.7%CV; intrasample, 0.42%CV); hence, it was used to calculate the relative expression of target genes. Relative expression was calculated by determining the difference of Ct between the genes of interest and the housekeeping gene (2^−ΔΔCt^), while arbitrarily considering CD-exposed male offspring as the reference group [54]. Results were presented in ratio fold, and statistical analysis was assessed on ΔΔCt which are normally distributed.

**Table 1:** Primers sequence for rt-qPCR experiments

Targets	Primer 5′	Primer 3′
***Nfκb***	CAC CTA GCT GCC AAA GAA GG	GCA GGC TAT TGC TCA TCA CA
***Cox2***	TGF TGG TGG AAA AAC CTC GT	GGT GCT CGG CTT CCA GTA TT
***Tmem119***	TTC TTC CGG CAG TAC GTG AT	CGA GGA TGG GTA GTA GGC TG
***Aif1***	TCT GCC GTC CAA ACT TGA AG	GCC ACT GGA CAC CTC TCT AA
***Trem2***	ACC CTC TAG ATG ACC AAG ATG C	TTG GGC ACC CTC GAA ACT C
***Cx3cr1***	CAA GCT CAC GAC TGC CTT CT	TGT CCG GTT GTT CAT GGA GTT
***Tgfb1***	ACA TGT GGA ACT CTA CCA GAA A	CTG CCG TAC AAC TCC AGT GA
***Rpl32***	TTG TTG CTC CCA TAA CCG ATG	TTA AGC GAA ACT GGC GGA AAC
***Gapdh***	GGA GAA ACC TGC CAA GTA TGA	GGT CCT CAG TGT AGC CCA AG

*Aif1* allograft inflammatory factor 1, *Cox2* cyclooxygenase 2, *Cx3cr1* fractalkine receptor, *Gapdh* glyceraldehyde 3-phosphate dehydrogenase, *Nfκb* nuclear factor kappa B, *Tgfb1* transforming growth factor β1, *Tmem119* transmembrane protein 119, *Trem2* triggering receptor expressed by myeloid cells 2

Other than the cytokine *Tgfb1*, we attempted to assess mRNA levels of *Il1b* and *Il6*; however, we failed to obtain robust amplification by rt-qPCR during the primer design phase.

### Microglial density, distribution, and morphology analysis

PFA-perfused brain sections were double-immunostained against IBA1 (labels all myeloid cells including microglia) and transmembrane protein 119 (TMEM119; microglia-specific [55]) to evaluate microglial density, distribution, and morphology, as well as myeloid cell infiltration as previously described [56]. Three to four brain sections containing the dorsal hippocampus CA1 (Bregma, − 1.31 to − 1.91; stereotaxic atlas of Paxinos and Franklin 4th edition [57]) were selected for each offspring (*n* = 5 animals/sex/diet). Sections were washed, incubated 40 min in sodium citrate buffer to expose epitopes, washed again and treated with 0.1% NaBH_4_ (cat#480886, MilliporeSigma) to quench autofluorescence. Afterwards, brain sections were incubated in blocking solution (0.5% gelatin, 5% donkey serum, 0.1% Triton X-100) for 1 h at room temperature, washed and incubated overnight at 4 °C with a cocktail of monoclonal primary antibodies in blocking solution: mouse anti-IBA1 (1:190; cat# MABN92, Millipore Sigma) and rabbit anti-TMEM119 (1:300; cat# ab209064, Abcam). The sections were washed and incubated with the polyclonal secondary antibodies donkey anti-mouse Alexa555-conjugated (1:300 in PBS; cat#A31570, Invitrogen, ThermoFisher Scientific) and donkey anti-rabbit Alexa647-conjugated (1:300 in PBS; cat#A31573, Invitrogen, ThermoFisher Scientific) for 1.5 h at room temperature. Sections were then mounted on slides and coverslipped in Fluoromount mounting medium (cat# 0100-01, SouthernBiotech, Birmingham, AB, USA).

For density and distribution analysis, all stained sections were imaged in a single plane mosaic at × 20 using an Axio Imager M2 epifluorescence microscope equipped with an AxioCam MRm camera and acquired with the Zen Pro 2012 software (Zeiss, Oberkochen, Germany). For morphology analysis, z-stacks (*stratum radiatum* (*st rad*), 18–20 z-stacks/animal; *stratum lacunosum moleculare* (*st lac mol*), 10–15 z-stacks/animal) were captured at × 40 using a Quorum Wave FX spinning disc confocal microscope (Quorum Technologies, Guelph, ON, Canada) equipped with an ORCA-R2 camera (512 × 512 pixels; Hamamatsu Photonics, Hamamatsu, Japan). The z-stacks were merged into a single plane using the Volocity software (Version 5.4, PerkinElmer, Waltham, MA, USA).

All analyses were performed blind to the experimental condition using the ImageJ software (v1.51j8; National Institute of Health, Bethesda, MD, USA). Total count of IBA1^+^/TMEM119^+^ (microglia) and IBA1^+^/TMEM119^−^ (peripheral macrophages) cells was compiled for the dorsal hippocampus CA1, *st rad* and *st lac mol*, across 6–8 hippocampi per animal, using the analyze particles plugin [56, 58]. Afterwards, cellular distribution was assessed using the nearest neighbor distance (NDD) plugin. For microglial morphology analysis, the number of branches and junctions, as well as average and longest branch length, was obtained (*st rad*: *n* = 20–25 microglia/animal, *st lac mol*: *n* = 15–20 microglia/animal, *n* = 5 animals/sex/diet) using a semi-automatic method adapted from [59]. For each microglial cell, soma and manual arbor were also traced using the freehand tool and polygon selection tool to obtain area values. Morphological index for each microglia was calculated by dividing the soma area by the manual arborization area to help identify microglial changes from their steady state [56, 58]. Manual arbor selection was further processed in a semi-automated manner to obtain unsharp mask of the cell, adjusted by the observer when needed, and area of the cell as well as shape descriptor (i.e., circularity, solidity, and aspect ratio) values were measured. Circularity was calculated by 4π×(area/perimeter^2^), for which a value of 1.0 represents a perfect circle and towards 0.0 an elongated shape. Solidity was calculated by dividing the cell area by the convex cell area meaning that a value close to 0.0 indicates a porous shape and close to 1.0 a convex shape. Finally, the aspect ratio was calculated by dividing the major axis of the cell by the minor axis of the cell, meaning a value of 1.0 similar ratio of minor and major axis and the higher the value the more elongated the cell is. The mask of the cell was skeletonized and analyzed using skeleton 2D/3D plugin. Skeleton analysis allowed us to determine number, average length, and maximal length of branches as well as number of junctions.

### Microglial ultrastructure analysis

Two PFA/acrolein-perfused brain sections containing the dorsal hippocampus CA1 (Bregma − 1.67 mm; stereotaxic atlas of Paxinos and Franklin 4th edition [57]) were selected in each of four animals per group. Sections were washed in PBS, then quenched 10 min in 0.3% H_2_O_2_ in PBS and permeabilized 30 min in 0.1% NaBH_4_ in PBS. Sections were first incubated 1 h at room temperature in blocking solution (10% fetal bovine serum, 3% bovine serum albumin, 0.01% Triton X-100 in [50 mM] tris-buffered saline (TBS)). Afterwards, sections were incubated overnight at 4°C with rabbit anti-IBA1 polyclonal primary antibody (1:1000; cat#019-19741, FUJIFILM Wako Chemical, Osaka, Japan) in blocking solution. The following day, antibody was washed out, and the sections were incubated with biotinylated goat anti-rabbit polyclonal secondary antibody (cat# 111-066-046, Jackson ImmunoResearch, West Grove, PA, USA) in TBS for 1.5 h, followed by avidin-biotin complex solution (1:1:100 in TBS; cat# PK-6100, Vector Laboratories, Burlingame, CA, USA) for 1 h at room temperature. The staining was revealed in 0.05% diaminobenzidine (DAB; cat# D5905-50TAB, Millipore Sigma) with 0.015% H_2_O_2_ in TBS for 4.5 min at room temperature.

The immunostained sections were next post-fixed flat in osmium-thiocarbohydrazide-osmium for scanning electron microscopy (SEM). In particular, sections were incubated in 3% ferrocyanide (cat# PFC232.250, BioShop, Burlington, ON, Canada) diluted in water combined (1:1) with 4% aqueous osmium tetroxide (cat#19170, Electron Microscopy Sciences, Hatfield, PA, USA) for 1 h, in 1% thiocarbohydrazide diluted in water (cat# 2231-57-4, Electron Microscopy Sciences) for 20 min, in 2% osmium tetroxide diluted in water, then dehydrated in ascending concentration of ethanol (2 × 35%, 50%, 70%, 80%, 90%, 3 × 100%) followed by propylene oxide (3×) for 5 min each. After post-fixation, tissues were embedded in Durcupan ACM resin (cat# 44611-44614, Millipore Sigma) for 24 h and carefully placed between two ACLAR® embedding films (cat# 50425-25, Electron Microscopy Sciences), and the resin was let to polymerize at 55 °C for 72 h. Regions of selection—dorsal hippocampus CA1—were excised from the embedded sections on ACLAR® sheets and re-embedded on top of a resin block for ultrathin sectioning (Ultracut UC7 ultramicrotome, Leica Biosystems). Ultrathin sections (~ 75 nm thickness) were collected and placed on a silicon nitride chip and glued on specimen mounts for SEM. Seven to 12 microglial cell bodies in each animal/layer of interest (*st rad* and *st lac mol*) were imaged at 5 nm of resolution using a Crossbeam 540 field emission SEM with a Gemini column (Zeiss).

Ultrastructural analysis was performed blind to the experimental conditions using the ImageJ software (*st rad*: *n* = 32–41 microglia/sex/diet, *st lac mol*: *n* = 28–36 microglia/sex/diet, *n* = 4 animals/sex/diet). Microglial endoplasmic reticulum, Golgi apparatus, lysosomes, lipofuscin, mitochondria, and endosomes were first analyzed quantitatively [60]. Dilation of the endoplasmic reticulum and/or Golgi apparatus was noted when the distance between the cisternal membranes was 50 nm or greater [61, 62]. Lysosomes were identified by their dense heterogenous contents enclosed by a single membrane [63, 64]. Secondary lysosomes were differentiated from primary lysosomes by their contacts with fusion endosomes. Tertiary lysosomes were identified by their contacts with lipofuscin and often also with fusion endosomes [63, 65]. Lipofuscin granules, for their part, were identified by their oval or round structure and finely granular composition with a unique fingerprint-like pattern [65]. Mitochondria were considered as elongated when their length was greater than 1 μm [60]. Microglial contacts with the cell bodies from other brain cells (i.e., astrocytes, neurons, oligodendrocytes) as well as blood vessels were quantified. For the neurons, contacted myelinated axons and synaptic elements—pre-synaptic axon terminal or post-synaptic dendritic spine—were further identified. Astrocytic cells were identified by their pale nuclei with a thin rim of heterochromatin and pale irregular cytoplasm, often containing intermediary filaments [66]. Neurons were distinguished by their pale nuclei and pale cytoplasm, often with an apical dendrite and innervation from axon terminals [66]. Pre-synaptic axon terminals were differentiated by their synaptic vesicles, while post-synaptic spines were in contact with a pre-synaptic axon terminal, often with a visible post-synaptic density at their junction [66]. Microglia were recognized by their dark irregular nuclei with a heterogenous chromatin pattern and a dark irregular cytoplasm, often containing short endoplasmic reticulum cisternae and lipidic inclusions (i.e., lipofuscin, lipid bodies or droplets, lysosomes) [66]. Similar to microglia, oligodendrocytes were identified by their dark nuclei with a heterogenous chromatin pattern and dark squarish or rectangular-shape cytoplasm, often containing short and wide endoplasmic reticulum cisternae organised in the vicinity of the nucleus and ribosomes, as well as a wider space between nuclear membranes than microglia [66]. In the vicinity of microglia, the occurrence of degradation activities (degenerating myelin, extracellular digestion) was also noted. Extracellular digestion, also named “exophagy,” was identified as extracellular space pockets containing degraded elements or debris [67, 68]. In contrast, degenerating myelin was recognized by ballooning, swelling, or distancing of myelin sheaths [66].

In addition, the density of dark cells and apoptotic cells was assessed (*n* = 4 animals/sex/diet). Then, dark cells were analyzed in a semi-quantitative manner (*st rad*: *n* = 0–6 dark cells/sex/diet, *st lac mol*: *n* = 13–19 dark cells/sex/diet, *n* = 4 animals/sex/diet). Dark cells were distinguished by their electron-dense nuclei showing a loss of the chromatin pattern and electron-dense cytoplasm presenting several signs of cellular stress (i.e., dilated endoplasmic reticulum and Golgi apparatus cisternae, elongated mitochondria) [69]. In the present study, we distinguished between two types of dark cells: microglia and perivascular cells. In addition to their dark features, dark microglia were recognised by their microglial characteristics [60, 69] and were located inside the brain parenchyma. Dark perivascular cells were identified by their localization enclosed in the perivascular space and possessed the dark features mentioned above. Apoptotic cells were also dark and recognised with their pyknotic nucleus and accumulation of autophagic endosomes [70].

Sample size of dark cells in *st lac mol* (*n* = 59 individual dark cells total) was considered sufficient to attempt statistical data analysis. This assumption was based on the sample size calculated using the G*Power software (v3.1.9.6) [71] to detect a large effect size of 0.4 that was estimated to 52 individuals cells.

### Statistical analyses

Data are reported as means ± standard error of the mean (SEM). Sample size (*n*) refers to individual animals for metabolic parameters, immune priming, gene expression as well as microglial density and morphology analyses, while it refers to individual microglia or dark cells for ultrastructural analyses. Statistical analyses were conducted using Prism 8 (v.8.3, GraphPad Software, San Diego, CA, USA). Normality was verified using Shapiro-Wilk and assessed by QQ plot. For normally distributed dataset, Grubbs’ test (two-tailed, *α* = 0.05) was used to identify outliers that were removed from the datasets prior to performing parametric statistic tests. To compare CD vs HFD in the dams, a Student *t* test was used for non-repeated measures including glucose levels, weight, and fat deposit measurements, while a 2-way analysis of variance (ANOVA) for repeated measures test was used for comparing weight and dietary follow-up data across time. In the offspring, a 2-way ANOVA was used to compare CD vs mHFD, as well as male and female animals for metabolic parameters (i.e., weight and fat deposits), gene expression, as well as microglial density, distribution, morphology, and ultrastructure. Significant ANOVA tests with a sex × diet interaction were followed by Bonferroni post-hoc test to identify significant differences between individual groups. For non-normally distributed dataset, a Mann-Whitney test was used to compare CD vs HFD in the dams for cytokines profile, gestation duration, and litter size. For non-normally distributed offspring dataset, a mixed-effect model was used to compare CD vs mHFD, males vs females in terms of myeloid cells infiltration, as well as to compare SAL vs LPS groups after immune challenge to assess cytokines profile. Significant mixed-effect model was followed by a Bonferroni post-hoc test. Statistically significant differences were considered for *p* value < 0.05.

## Results

### High-fat diet induces long-term increase of peripheral IL-6 and fat deposits in dams and male offspring

HFD has been associated with a variety of metabolic changes including increased body fat, obesity, diabetic-like phenotype (glucose and/or insulin intolerance) [72], and decreased fertility [73]. To characterize further our model, we assessed weight and food consumption, glucose levels, fat deposits, as well as gestation duration and litter size in the dams. These measurements revealed that mHFD does not induce obesity, although it is associated with an overconsumption of fats to the detriment of carbohydrates (Supplementary Figure 1) and does not induce glycemia changes (Supplementary Figure 2 a). HFD nevertheless resulted in an increase of retroperitoneal, subcutaneous, and perigonadal fat deposition at the measured endpoint, without driving an overall increase of total body mass or an obese phenotype (Supplementary Figure 2 b-g). Importantly, it did not lead to fertility alterations, ruling out major metabolic alterations as often seen in diet-induced animal models of obesity (Supplementary Figure 2 h-k).

 Studying offspring exposed to a mHFD revealed that they are more prone to developing metabolic syndrome, which includes increase in fat deposits and body weight [2]. In our study, mHFD offspring had similar body weight compared to CD offspring at PND30, but the mHFD males showed increased perigonadal fat deposits compared to CD males (Supplementary Figure 3), which highlights a sexually dimorphic effect of mHFD on offspring fat deposition.

**Fig. 2:**
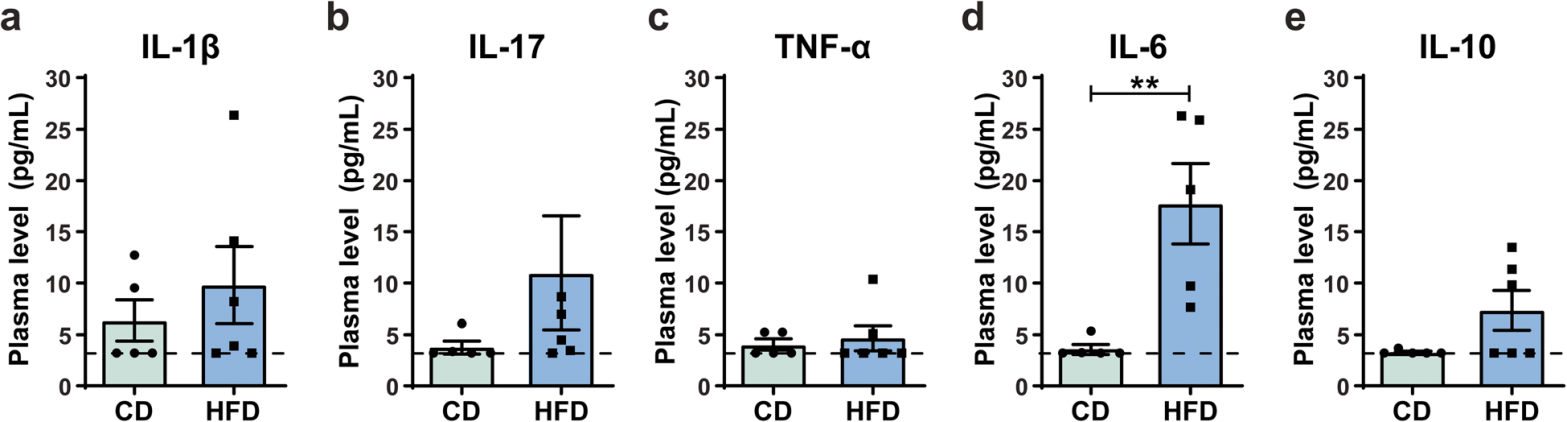
HFD effect on peripheral inflammatory profile in the dams at weaning of their litters. Plasma levels were measured by multiplex-ELISA for **a** IL-1 β, **b** IL-17, **c** TNF-α, **d** IL-6, and **e** IL-10. Detection limit of each graphs is represented by a dotted line. Data are shown as mean ± standard error of the mean. *p* < 0.01** by Mann-Whitney test. CD, control diet; HFD, high-fat diet; IL, interleukin; TNF-α, tumor necrosis factor α

Rodent models of mHFD were also previously shown to induce peripheral inflammation, notably by increasing the levels of cytokines in maternal blood circulation [14, 28, 74, 75]. To evaluate the maternal immune profile in our mouse model of mHFD, we measured plasma levels of pro- (IL-1β, IL-6, IL-17, TNF-α) and anti-inflammatory (IL-10) cytokines in the dams at weaning of their litter by multiplex-ELISA. Plasma levels of IL-6 were significantly increased (*U* = 0, n_1_ = n_2_ = 5, *p =* 0.0079) in HFD-fed dams (17.74 ± 8.79 pg/mL) compared to CD-fed dams (3.636 ± 0.933 pg/mL) (Fig. 2d), while both diet groups had similar levels of IL-1 β, IL-10, IL-17, and TNF-α (Fig. 2a–b, e). Overall, these results suggest a MIA phenotype induced by mHFD in our model, confirmed by the increase of IL-6.

### mHFD offspring have higher IL-6 plasma levels after LPS-induced immune challenge, while hippocampal inflammatory response remained similar to CD-exposed offspring

**Fig. 3:**
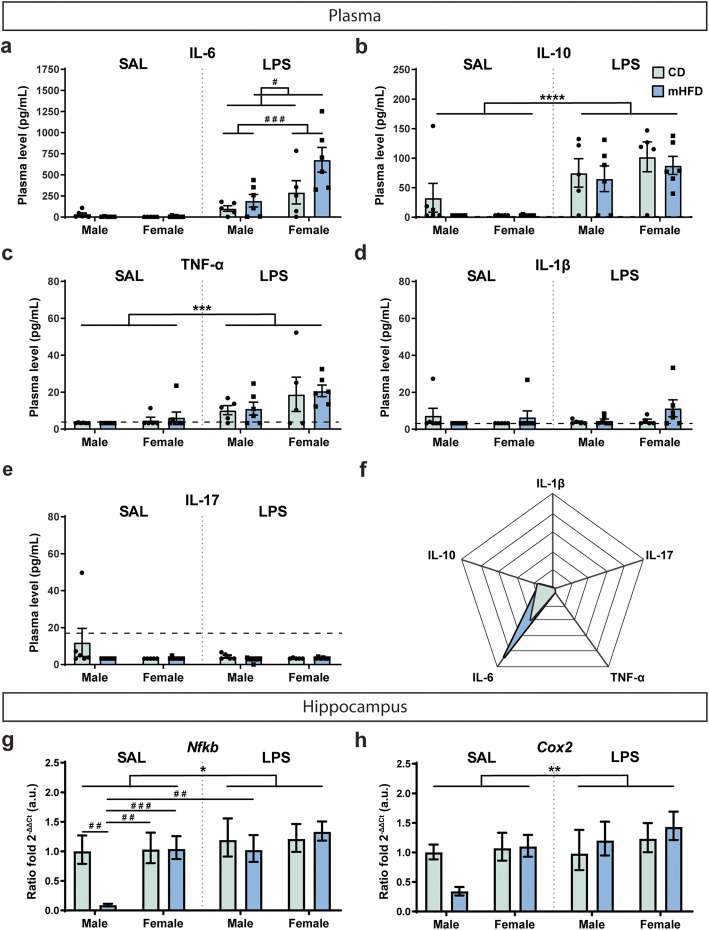
mHFD effect on plasma cytokine profile and brain inflammatory profile of PND30 offspring 8 h after LPS treatment. **a** IL-1β, **b** IL-17, **c** TNF-α, **d** IL-6, and **e** IL-10 were measured by multiplex-ELISA in offspring plasma after administration of SAL or LPS by intraperitoneal injection. Dotted line on the graphs indicates the detection limits. **f** Radar graph illustrates cytokines levels in offspring from both diet groups regardless of their sex 8 h after LPS immune challenge. **g** *Nfκb* and **h** *Cox2* mRNA levels were evaluated by rt-qPCR in offspring hippocampus after administration of SAL or LPS by intraperitoneal injection. Data are shown as mean ± standard error of the mean. *p* < 0.05*, *p* < 0.001***, *p* < 0.0001**** (treatment) by mixed-effects analysis, *p* < 0.05^#^ (treatment × diet), *p* < 0.01^#^ (treatment × sex × diet), *p* < 0.001 ^# # #^ (treatment × sex or treatment × sex × diet) by mixed-effects analysis. CD, control; *Cox2*, *Cyclooxygenase 2*; LPS, lipopolysaccharide; IL, interleukin; SAL, saline; mHFD, maternal high-fat diet; *Nfκb*, *nuclear factor kappa B*; TNF-α, tumor necrosis factor α

To assess peripheral immune priming in the adolescent offspring, we measured circulating cytokines by multiplex ELISA at PND30, 8 h after systemic injection of low dose LPS vs SAL. This timepoint corresponds to a period of inflammatory resolution after the immune challenge in adult mouse brain, allowing us to evaluate prolonged inflammatory response [50–53]. In LPS-injected animals, plasma levels of IL-6 were significantly elevated (*F*_(1,37)_ = 10.44, *p* = 0.0317) in mHFD vs CD offspring regardless of their sex (436.8 ± 107.0 pg/mL vs 197.9 ± 74.1 pg/mL) (Fig. 3a). Moreover, regardless of their maternal diet, LPS-treated female offspring had significantly (*F*_(1,37)_ = 5.324, *p* = 0.0004) increased plasma levels of IL-6 compared to LPS-treated male offspring (504.1 ± 113.8 pg/mL vs 152.3 ± 44.0 pg/mL) (Fig. 3a). Levels of TNF-α and IL-10 were also significantly increased (TNF-α: *F*_(1,37)_ = 16.43, *p* = 0.0002; IL-10: *F*_(1,21)_ = 30.63, *p* < 0.0001) in LPS-treated offspring compared to SAL-treated offspring regardless of their sex or maternal diet (TNF-α, 15.23 ± 2.66 pg/mL vs 4.435 ± 0.745 pg/mL; IL-10, 82.54 ± 8.03 pg/mL vs 10.76 ± 7.40 pg/mL) (Fig. 3b–c, f). Finally, levels of IL-1β and IL-17 were similar between SAL-treated and LPS-treated offspring regardless of their sex and maternal diet (Fig. 3d–f). Together, these results indicate that systemic LPS administration induced IL-6, IL-10, and TNF-α release detected 8 h afterwards in CD and mHFD offspring, while IL-6 release was significantly exacerbated in mHFD offspring, indicating either a sustained inflammation or a stronger response to the immune challenge specific to this cytokine upon exposure to mHFD.

Similarly, we evaluated inflammatory response in the hippocampus by measuring mRNA transcript expression by rt-qPCR of two genes downstream of pro-inflammatory cytokines during inflammation, *Nfκb* [76, 77] and *Cox2* [51], 8 h after administration of LPS or SAL*.* Upon LPS treatment, both CD- and mHFD-exposed offspring showed significantly increased mRNA expression level of *Nfκb* (SAL, 0.711 vs LPS, 1.184 ratio fold; *F*_(1,37)_ = 5.646, *p* = 0.0228; detailed in Table 2; Fig. 3g) and *Cox2* (SAL, 0.793 vs LPS, 1.200 ratio fold; *F*_(1,37)_ = 7.608, *p* = 0.0090; detailed in Table 2; Fig. 3h). In the SAL condition, *Nfκb* level was decreased in mHFD-exposed male offspring compared to other offspring groups (*F*_(1,37)_ = 5.344, SAL-CD male: *p* = 0.0044, SAL-CD female: *p* = 0.0037, SAL-mHFD female: *p* = 0.0002; detailed in Table 2; Fig. 3g). Taken together, these results suggest that immune response of the hippocampus was similar with mHFD. However, *Nfκb* homeostatic functions (e.g., synaptic plasticity and regulation of neuronal excitability) [77] may be altered at steady-state in mHFD-exposed male offspring.

**Table 2:** mHFD effect on hippocampal mRNA levels of PND30 offspring

Gene	Treatment	Male		Female		***F***	***p***
		CD	mHFD	CD	mHFD		
***Nfκb***	**SAL**	2^−ΔΔCt^ = 1.000 ΔΔCt = 0.000 ±0.344	2^−ΔΔCt^ = 0.090 ΔΔCt = 2.633 ± 0.833	2^−ΔΔCt^ = 1.030 ΔΔCt = 0.038 ± 0.360	2^−ΔΔCt^ = 1.040 ΔΔCt = 0.630 ± 0.569	Treatment × sex × diet, 5.344 Treatment × sex, 5.513 Treatment × diet, 2.523 Sex × diet, 8.428 Treatment, 5.646 Sex, 8.928 Diet, 2.926	Treatment × sex × diet, 0.0265* Treatment × sex, 0.0243* Treatment × diet, 0.1207 Sex × diet, 0.0062** Treatment, 0.0228* Sex, 0.0050** Diet, 0.0955
	**LPS**	2^−ΔΔCt^ = 1.190 ΔΔCt = 0.255 ± 0.385	2^−ΔΔCt^ = 1.020 ΔΔCt = 0.034 ± 0.317	2^−ΔΔCt^ = 1.210 ΔΔCt = 0.270 ± 0.281	2^−ΔΔCt^ = 1.330 ΔΔCt = 0.415 ± 0.175		
***Cox2***	**SAL**	2^−ΔΔCt^ = 1.000 ΔΔCt = 0.000 ± 0.182	2^−ΔΔCt^ = 0.340 ΔΔCt = 1.574 ± 0.312	2^−ΔΔCt^ = 1.070 ΔΔCt = 0.101 ± 0.324	2^−ΔΔCt^ = 1.100 ΔΔCt = 0.133 ± 0.242	Treatment × sex × diet, 3.711 Treatment × sex, 2.052 Treatment × diet, 5.596 Sex × diet, 3.170 Treatment, 7.608 Sex, 7.507 Diet, 1.419	Treatment × sex × diet, 0.0618 Treatment × sex, 0.1604 Treatment × diet, 0.0233* Sex × diet, 0.0832 Treatment, 0.0090** Sex, 0.0094** Diet, 0.2412
	**LPS**	2^−ΔΔCt^ = 0.980 ΔΔCt = 0.022 ± 0.490	2^−ΔΔCt^ = 1.200 ΔΔCt = 0.264 ± 0.342	2^−ΔΔCt^ = 1.230 ΔΔCt = 0.293 ± 0.289	2^−ΔΔCt^ = 1.430 ΔΔCt = 0.516 ± 0.242		
***Tgfb1***	–	2^−ΔΔCt^ = 1.000 ΔΔCt = 0.000 ± 0.218	2^−ΔΔCt^ = 0.2764 ΔΔCt = 1.860 ± 0.262	2^−ΔΔCt^ = 1.139 ΔΔCt = − 0.190 ± 0.407	2^−ΔΔCt^ = 1.282 ΔΔCt = − 0.360 ± 0.335	Sex × diet, 10.05 Sex, 14.16 Diet, 6.965	Sex × diet, 0.0050** Sex, 0.0013** Diet, 0.0162*
***Aif1***	–	2^−ΔΔCt^ = 1.000 ΔΔCt = 0.000 ± 0.328	2^−ΔΔCt^ = 0.7169 ΔΔCt = 0.480 ± 0.340	2^−ΔΔCt^ = 1.069 ΔΔCt = − 0.100 ± 0.290	2^−ΔΔCt^ = 1.196 ΔΔCt = − 0.260 ± 0.299	Sex × diet, 1.035 Sex, 1.783 Diet, 0.2588	Sex × diet, 0.3218 Sex, 0.1976 Diet, 0.6168
***Tmem119***	–	2^−ΔΔCt^ = 1.000 ΔΔCt = 0.000 ± 0.313	2^−ΔΔCt^ = 0.1346 ΔΔCt = 2.89 ± 0.269	2^−ΔΔCt^ = 1.260 ΔΔCt = − 0.330 ± 0.350	2^−ΔΔCt^ = 1.101 ΔΔCt = − 0.140 ± 0.265	Sex × diet, 19.47 Sex, 30.15 Diet, 25.34	Sex × diet, 0.0003*** Sex, < 0.0001**** Diet, < 0.0001****
***Trem2***	–	2^−ΔΔCt^ = 1.000 ΔΔCt = 0.000 ± 0.282	2^−ΔΔCt^ = 0.1690 ΔΔCt = 2.570 ± 0.229	2^−ΔΔCt^ = 0.9781 ΔΔCt = 0.030 ± 0.326	2^−ΔΔCt^ = 0.9598 ΔΔCt = 0.060 ± 0.242	Sex × diet, 20.88 Sex, 19.90 Diet, 21.88	Sex × diet, 0.0002*** Sex, 0.0003*** Diet, 0.0002***
***Cx3cr1***	–	2^−ΔΔCt^ = 1.000 ΔΔCt = 0.000 ± 0.203	2^−ΔΔCt^ = 0.1724 ΔΔCt = 2.520 ± 0.479	2^−ΔΔCt^ = 1.147 ΔΔCt = − 0.200 ± 0.315	2^−ΔΔCt^ = 1.207 ΔΔCt = − 0.270 ± 0.276	Sex × diet, 16.44 Sex, 21.92 Diet, 14.71	Sex × diet, 0.0007*** Sex, 0.0002*** Diet, 0.0011**

Transcripts level of *Nfκb*, Cox2, *Tgf1b*, *Aif1*, *Tmem119*, *Trem2*, and *Cx3cr1* were normalized by *Rpl32*, where SAL-CD male or CD male offspring represent the reference group. *Nfκb* and *Cox2* were measured 8 h after LPS-induced immune challenge, while *Tgf1b*, *Aif1*, *Tmem119*, *Trem2*, and *Cx3cr1* were evaluated in homeostatic condition.

*2*^*−ΔΔCt*^ ratio fold compared to expression of the reference group, *ΔΔCt* difference of cycle threshold between ΔCt of the target gene and ΔCt of *Rpl32*, *Aif1* allograft inflammatory factor 1, *CD* control diet, *Cox2* cyclooxygenase 2, *Cx3cr1* fractalkine receptor, *LPS* lipopolysaccharide, *mHFD* maternal high-fat diet, *Nfκb* nuclear factor kappa B, *SAL* saline, *Tgfb1* transforming growth factor β1, *Tmem119* transmembrane protein 119, *Trem2* triggering receptor expressed by myeloid cells 2, *P*-values of significant statistical tests are in bold and followed by the number of asterisks indicating their significativity, *p* < 0.05*, *p* < 0.01**, *p* < 0.001***, *p* < 0.0001****

### Male offspring have altered gene expression after mHFD whereas both male and female offspring exhibit altered microglial morphology

**Fig. 4:**
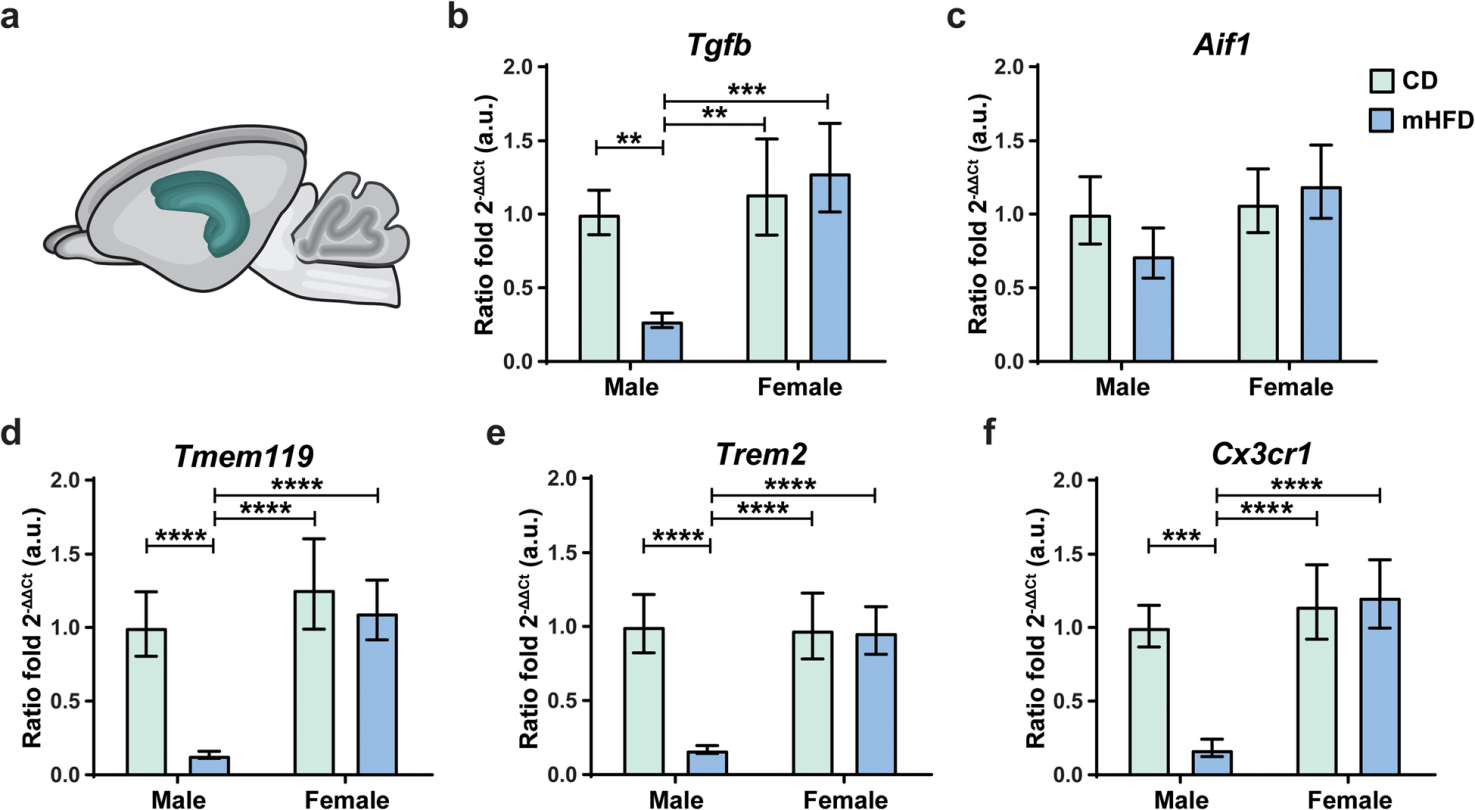
mHFD effect on microglia-related mRNA expression in the hippocampus of PND30 offspring. **a** Scheme illustrating the region of interest, the hippocampus, in sagittal and coronal views of a mouse brain. mRNA expression (2^-ΔΔCt­) was evaluated for *Tgfb1* (**b**), *Aif1* (**c**), *Tmem119* (**d**), *Trem2* (**e**), and *Cx3cr1* (**f**) normalized by housekeeping gene *Rpl32*. Data are shown as 2^(-ΔΔCt­ _Mean_ ± standard error of the mean). *p* < 0.001**, *p* < 0.001***, *p* < 0.0001**** (sex × diet) by 2-way ANOVA followed by Bonferroni post-hoc test. *Aif1*, allograft inflammatory factor 1; a.u., arbitrary units; CD, control; Ct, cycle threshold; *Cx3cr1*, fractalkine receptor; mHFD, maternal high-fat diet; *Tgfb1*, transforming growth factor β1; *Tmem119*, transmembrane protein 119; *Trem2*, triggering receptor expressed by myeloid cells 2

Other than a peripheral inflammatory response, immune priming has been associated with changes in gene expression and/or morphology of immune cells—including microglia in the brain [1, 28, 78]. To characterize microglia-related gene changes, we used rt-qPCR to study mRNA expression in whole hippocampus of mHFD vs CD offspring at PND30 (Fig. 4a). We focused on *Tgfb1*—a cytokine that modulates inflammation and microglia survival [79]—as well as on receptors mainly expressed by microglia in the brain that are involved with the regulation of inflammation (i.e., *Aif1* [80]), microglial survival (i.e., *Tmem119* [55], *Trem2* [81]), or synaptic remodeling (i.e., *Cx3cr1* [82–84], *Trem2* [82, 85–87]). mHFD-exposed male offspring had significantly reduced expression of *Tgfb1* (*F*_(1,19)_ = 10.05, CD male: *p* = 0.0045, CD female: *p* = 0.0018, mHFD female: *p* = 0.0008) compared to other offspring groups (detailed in Table 2; Fig. 4b). Expression of *Aif1* was however similar between groups (Fig. 4c). mHFD-exposed male offspring also had a significantly lower ratio fold of *Tmem119* (*F*_(1,19)_ = 19.47, CD male: *p* < 0.0001, CD female: *p* < 0.0001, mHFD female: *p* < 0.0001), *Trem2* (*F*_(1,19)_ = 20.88, CD male: *p* < 0.0001, CD female: *p* < 0.0001, mHFD female: *p* < 0.0001), and *Cx3cr1* (*F*_(1,19)_ = 16.44, CD male: *p* = 0.0002, CD female: *p* < 0.0001, mHFD female: *p* < 0.0001) compared to the other offspring groups (detailed in Table 2; Fig. 4d–f). Together, these results indicate that mHFD leads to altered expression, specifically in males, of inflammatory-regulating (*Tgfb1*) as well as microglial function-related (*Tmem119*, *Trem2*, and *Cx3cr1*) genes.

**Fig. 5:**
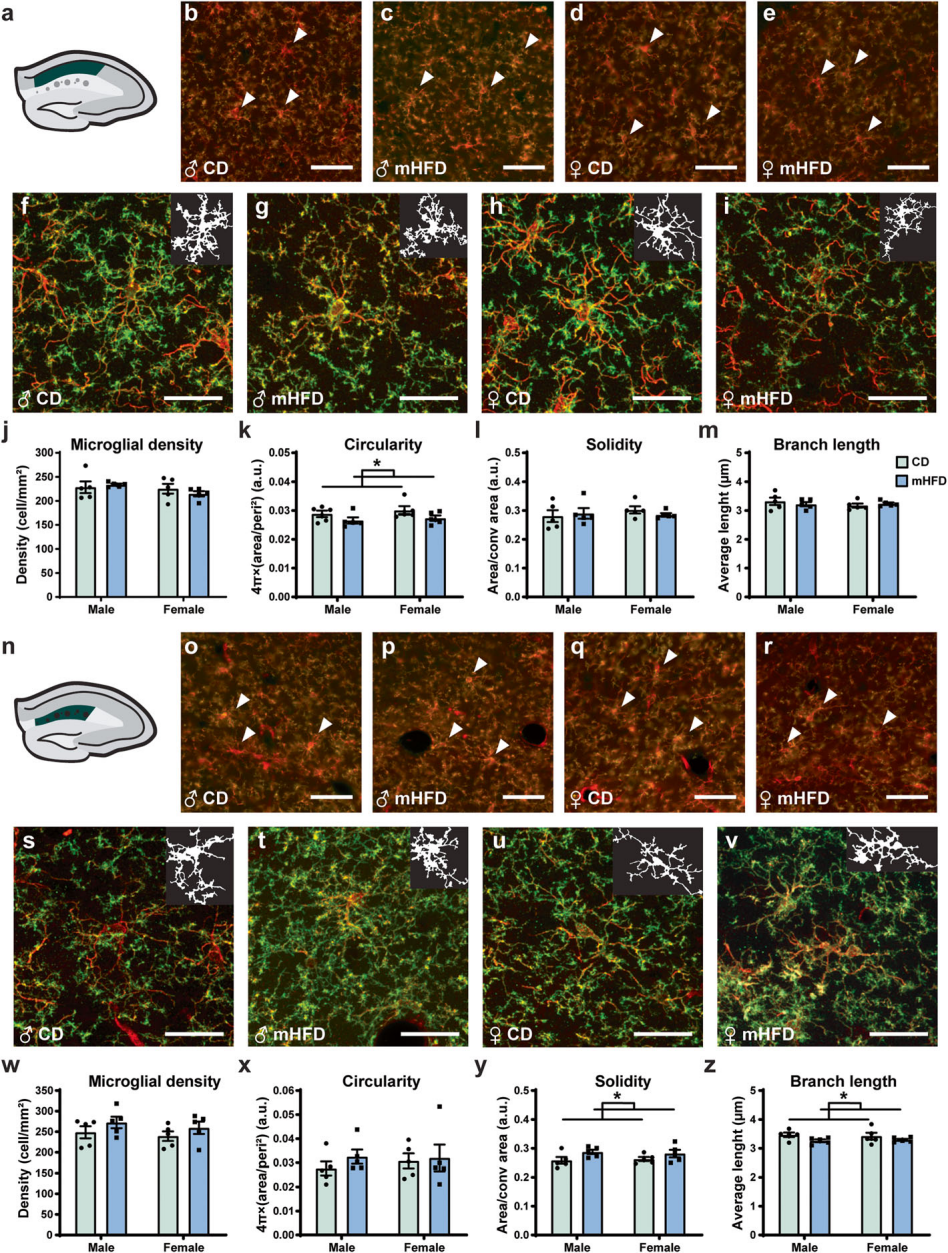
mHFD effect on microglial density, distribution, and morphology in the dorsal hippocampus CA1 of PND30 offspring. Representative scheme of the **a** *st rad* and **n** *st lac mol* to illustrate the layers analyzed. Immunofluorescence (IBA1+ low). Immunofluorescence IBA1 (red) and TMEM119 (green) allowed analysis of (**b**–**e**, **o**–**r**) microglial number and morphology (**f**–**i**, **s**–**v**). Scale bar for the density pictures is equivalent to 50 μm (**b**–**e**, **o**–**r**), whereas scale bar for morphology pictures is 25 μm (**f**–**i**, **s**–**v**). For each morphology picture, mask of the cell (IBA1^+^ staining) is represented in the upper right corner. Only (**j**, **w**) microglial density and main differences in morphology (**k**, **x** circularity; **l**, **y** solidity; and **m**, **z** branch length average) are presented on the figure. Data are shown as mean ± standard error of the mean. *p <* 0.05* (diet) by 2-way ANOVA. ♀, female; ♂, male; CD, control; Conv area, convex area; mHFD, maternal high-fat diet; Peri, perimeter

After performing rt-qPCR, we characterized the density, distribution, morphology, and ultrastructure of microglia in mHFD vs CD-exposed offspring at PND30. We focused on the dorsal hippocampus CA1, particularly the *st rad* and *st lac mol*—two main layers where neuronal plasticity occurs during cognitive processes [88] and that are associated to behavioral deficits previously reported in mHFD animal models [8, 14–18]. In both layers (Fig. 5a–e, n–r), the density and distribution of microglia (IBA1^+^/TMEM119^+^) and infiltrated myeloid cells (IBA1^+^/TMEM119^–^) were similar between groups (Table 3; Fig. 5j, w). Of note, infiltrated myeloid cells were marginal, accounting for 0.207% of IBA1+ cells in the *st rad* and 0.240% in *st lac mol*. Regardless of their sex and maternal diet, adolescent offspring displayed similar values for microglial soma, arbor, and cell area, as well as morphological index (soma area/manual arborization area) in both CA1 *st rad* and *st lac mol* (Tables 4 and 5; Fig. 5f–i, s–v). In *st rad*, further analysis of “skeletonized” microglia revealed no significant difference between offspring groups in terms of number of branches, junctions, as well as average and maximal branch length (Table 4; Fig. 5m). However, shape descriptor analysis of microglia identified a significant decrease in their circularity value (*F*_(1,16)_ = 4.683, *p =* 0.0459) in mHFD offspring compared to controls (0.0265 ± 0.0004 vs 0.0295 ± 0.0006), but solidity and aspect ratio remained unchanged (Table 4; Fig. 5k–l). In *st lac mol*, microglia of mHFD-exposed offspring had significantly shorter branch length (*F*_(1,16)_ = 4.553, *p =* 0.0487) compared to CD-exposed offspring (3.276 ± 0.015 μm vs 3.442 ± 0.022 μm) (Table 5; Fig. 5z). In addition to their shorter branch length, microglia of mHFD-exposed offspring had a significantly increased solidity (*F*_(1,16)_ = 5.616, *p* = 0.0307) compared to CD offspring (0.2845 ± 0.0030 vs 0.2603 ± 0.0023), regardless of the sex (Table 5; Fig. 5y), which could indicate a difference in microglial arborization distribution and/or organization with mHFD. In this layer, microglial branch number, maximal branch length, junction number, circularity, and aspect ratio were also similar between groups. Together, these morphological changes align with a microglial priming hypothesis, in which mHFD alters microglial morphology.

**Table 3:** mHFD effects on microglial density, distribution, and peripheral myeloid cell infiltration in the dorsal hippocampus CA1 of PND30 offspring

Parameters		Mean ± standard error of the mean				***F***	***p***
		Male		Female			
		CD	mHFD	CD	mHFD		
*St rad*	Density (cells/mm^2^)	228.5 ± 12.1	233.8 ± 2.1	225.4 ± 10.1	214.9 ± 5.4	Sex × diet, 0.8838 Sex, 1.725 Diet, 0.09162	Sex × diet, 0.3611 Sex, 0.2075 Diet, 0.7660
	Spacing Index (a.u.)	0.452 ± 0.014	0.469 ± 0.006	0.456 ± 0.011	0.454 ± 0.013	Sex × diet, 0.6857 Sex, 0.2303 Diet, 0.4325	Sex × diet, 0.4198 Sex, 0.6378 Diet, 0.5201
	Cluster	0.261 ± 0.138	0.051 ± 0.031	0.182 ± 0.084	0.222 ± 0.091	Sex × diet, 1.759 Sex, 0.2359 Diet, 0.8177	Sex × diet, 0.2034 Sex, 0.6338 Diet, 0.3793
	%Infiltration	0.201 ± 0.131	0.150 ± 0.092	0.270 ± 0.143	0.000 ± 0.000	Sex × diet, 1.031 Sex, 0.1433 Diet, 2.241	Sex × diet, 0.3250 Sex, 0.7100 Diet, 0. 1539
*St lac mol*	Density (cells/mm^2^)	248.8 ± 14.5	272.7 ± 14.2	239.4 ± 11.8	259.6 ± 15.0	Sex × diet, 0.01730 Sex, 0.6481 Diet, 2.507	Sex × diet, 0.8970 Sex, 0.4326 Diet, 0.1329
	Spacing Index (a.u.)	0.473 ± 0.019	0.466 ± 0.004	0.442 ± 0.016	0.470 ± 0.022	Sex × diet, 1.105 Sex, 0.6448 Diet, 0.4185	Sex × diet, 0.3089 Sex, 0.4337 Diet, 0.5268
	Cluster	0.122 ± 0.062	0.179 ± 0.093	0.029 ± 0.029	0.147 ± 0.065	Sex × diet, 0.2179 Sex, 0.9011 Diet, 1.739	Sex × diet, 0.6469 Sex, 0.3566 Diet, 0.2058
	%Infiltration	0.073 ± 0.073	0.437 ± 0.151	0.233 ± 0.154	0.216 ± 0.091	Sex × diet, 2.421 Sex, 0.06043 Diet, 2.006	Sex × diet, 0.1393 Sex, 0.8089 Diet, 0.1758

*%Infiltration* average percentage of IBA1^+^/TMEM119^−^ cells on total myeloid cells count, *a.u.* arbitrary unit, *CD* control diet, *mHFD* maternal high-fat diet, *St lac* mol *stratum lacunosum moleculare*, *St rad stratum radiatum*

**Table 4:** mHFD effects on microglial morphological parameters in the dorsal hippocampus CA1 *stratum radiatum* of PND30 offspring

Parameters	Mean ± standard error of the mean				***F***	***p***
	Male		Female			
	CD	mHFD	CD	mHFD		
Soma area (μm^2^)	46.59 ± 1.92	46.88 ± 2.54	44.42 ± 1.38	45.82 ± 1.84	Sex × diet, 0.08109 Sex, 0.6758 Diet, 0.1842	Sex × diet, 0.7795 Sex, 0.4231 Diet, 0.6735
Arbor area (μm^2^)	1352.68 ± 154.93	1355.63 ± 112.22	1374.23 ± 160.96	1377.16 ± 129.59	Sex × diet, 4.378 × 10^−9^ Sex, 0.02340 Diet, 0.0004357	Sex × diet, > 0.9999 Sex, 0.8803 Diet, 0.9836
Morphological index (a.u.)	0.036 ± 0.003	0.035 ± 0.001	0.034 ± 0.003	0.035 ± 0.004	Sex × diet, 0.09893 Sex, 0.1851 Diet, 0.0001109	Sex × diet, 0.7572 Sex, 0.6728 Diet, 0.9917
Cell area (μm^2^)	425.49 ± 86.24	430.71 ± 55.12	473.40 ± 79.36	435.13 ± 49.86	Sex × diet, 0.09819 Sex, 0.1422 Diet, 0.05674	Sex × diet, 0.7581 Sex, 0.7110 Diet, 0.8148
Circularity (a.u.)	0.029 ± 0.001	0.027 ± 0.001	0.030 ± 0.001	0.027 ± 0.001	Sex × diet, 0.03614 Sex, 0.7875 Diet, 4.683	Sex × diet, 0.8516 Sex, 0.3880 Diet, 0.0459*
Solidity (a.u.)	0.281 ± 0.020	0.290 ± 0.018	0.302 ± 0.012	0.285 ± 0.006	Sex × diet, 0.7345 Sex, 0.2907 Diet, 0.07122	Sex × diet, 0.4041 Sex, 0.5972 Diet, 0.7930
Aspect ratio (a.u.)	1.666 ± 0.055	1.648 ± 0.053	1.633 ± 0.065	1.663 ± 0.069	Sex × diet, 0.1587 Sex, 0.02512 Diet, 0.008885	Sex × diet, 0.6956 Sex, 0.8761 Diet, 0.9261
# Branches	101.92 ± 21.70	104.47 ± 13.79	113.11 ± 17.14	102.23 ± 12.52	Sex × diet, 0.1622 Sex, 0.07200 Diet, 0.06261	Sex × diet, 0.6925 Sex, 0.7919 Diet, 0.8056
Average branch length (μm)	3.318 ± 0.127	3.218 ± 0.075	3.172 ± 0.071	3.237 ± 0.041	Sex × diet, 0.9630 Sex, 0.5690 Diet, 0.04635	Sex × diet, 0.3410 Sex, 0.4616 Diet, 0.8323
Longest branch (μm)	13.29 ± 0.24	13.26 ± 0.49	12.77 ± 0.21	13.99 ± 0.21	Sex × diet, 4.048 Sex, 0.1145 Diet, 3.750	Sex × diet, 0.0614 Sex, 0.7395 Diet, 0.0707
# Junctions	52.38 ± 12.05	53.49 ± 7.28	58.43 ± 9.08	52.28 ± 6.78	Sex × diet, 0.1616 Sex, 0.07164 Diet, 0.07779	Sex × diet, 0.6930 Sex, 0.7924 Diet, 0.7839

*#* number, *%* percentage on total myeloid cells, *a.u.* arbitrary unit, *CD* control diet, *mHFD* maternal high-fat diet, *P*-values of significant statistical tests are in bold and followed by an asterisk indicating the significativity, * < 0.05

**Table 5:** mHFD effects on microglial morphological parameters in the dorsal hippocampus CA1 *stratum lacunosum moleculare* of PND30 offspring

Parameters	Mean ± standard error of the mean				***F***	***p***
	Male		Female			
	CD	mHFD	CD	mHFD		
Soma area (μm^2^)	40.18 ± 1.74	42.45 ± 2.06	40.14 ± 0.92	40.64 ± 1.57	Sex × diet, 0.2961 Sex, 0.3230 Diet, 0.7283	Sex × diet, 0.5938 Sex, 0.5777 Diet, 0.4060
Arbor area (μm^2^)	988.76 ± 125.44	908.98 ± 111.46	974.04 ± 118.48	973.34 ± 157.09	Sex × diet, 0.09350 Sex, 0.03685 Diet, 0.09685	Sex × diet, 0.7637 Sex, 0.8502 Diet, 0.7597
Morphological index (a.u.)	0.043 ± 0.004	0.049 ± 0.005	0.044 ± 0.006	0.047 ± 0.009	Sex × diet, 0.04385 Sex, 7.575 × 10^−5^ Diet, 0.5447	Sex × diet, 0.8368 Sex, 0.9932 Diet, 0.4712
Cell area (μm^2^)	272.57 ± 46.35	272.50 ± 36.57	269.99 ± 40.25	283.11 ± 43.67	Sex × diet, 0.02479 Sex, 0.009177 Diet, 0.02427	Sex × diet, 0.8769 Sex, 0.9249 Diet, 0.8782
Circularity (a.u.)	0.028 ± 0.003	0.033 ± 0.003	0.031 ± 0.003	0.032 ± 0.006	Sex × diet, 0.2333 Sex, 0.1162 Diet, 0.6419	Sex × diet, 0.6357 Sex, 0.7377 Diet, 0.4348
Solidity (a.u.)	0.258 ± 0.012	0.287 ± 0.008	0.263 ± 0.007	0.282 ± 0.013	Sex × diet, 0.2641 Sex, 0.004823 Diet, 5.616	Sex × diet, 0.6144 Sex, 0.9455 Diet, 0.0307*
Aspect ratio (a.u.)	1.803 ± 0.089	1.787 ± 0.058	1.793 ± 0.078	1.752 ± 0.094	Sex × diet, 0.02490 Sex, 0.07486 Diet, 0.1260	Sex × diet, 0.8766 Sex, 0.7879 Diet, 0.7273
# Branches	72.83 ± 12.86	73.24 ± 9.70	73.90 ± 11.91	77.18 ± 12.65	Sex × diet, 0.01466 Sex, 0.04465 Diet, 0.02438	Sex × diet, 0.9052 Sex, 0.8353 Diet, 0.8779
Average branch length (μm)	3.464 ± 0.077	3.262 ± 0.048	3.420 ± 0.120	3.291 ± 0.037	Sex × diet, 0.2168 Sex, 0.008423 Diet, 4.553	Sex × diet, 0.6477 Sex, 0.9280 Diet, 0.0487*
Longest branch (μm)	13.61 ± 0.46	12.51 ± 0.55	12.96 ± 0.57	12.98 ± 0.61	Sex × diet, 1.024 Sex, 0.02989 Diet, 0.9634	Sex × diet, 0.3265 Sex, 0.8649 Diet, 0.3409
# Junctions	36.88 ± 6.99	37.01 ± 5.19	37.61 ± 6.37	39.29 ± 6.71	Sex × diet, 0.01468 Sex, 0.05614 Diet, 0.02034	Sex × diet, 0.9051 Sex, 0.8157 Diet, 0.8884

*#* number, *%* percentage on total myeloid cells, *a.u.* arbitrary unit, *CD* control diet, *mHFD* maternal high-fat diet, *P-values* of significant statistical tests are in bold and followed by an asterisk indicating the significativity, * < 0.05

### Microglia from mHFD male offspring show increased interactions with astrocytes whereas both male and female offspring have decreased extracellular space pockets

To provide insights into microglial functions, we further performed SEM analysis to reveal, at nanoscale resolution, possible changes in their organelles and intercellular relationships in the dorsal hippocampus CA1 of mHFD- vs CD-exposed offspring, upon sacrifice at PND30. We determined the number of microglial organelles involved in phagolysosomal activity (primary, secondary and tertiary lysosomes, lipofuscin, endosomes with or without content) and alterations to organelles that serve as markers of cellular stress (dilated cisternae of endoplasmic reticulum and Golgi apparatus, elongated and total mitochondria). We also evaluated microglial interactions with their microenvironment, particularly direct contacts with astrocytic cell bodies, neuronal cell bodies, axon terminals, dendritic spines, oligodendrocytic cell bodies, myelinated axons, and blood vessels, as well as associations with extracellular space pockets containing degraded elements or debris indicative of extracellular digestion or “exophagy” [67, 68].

**Fig. 6:**
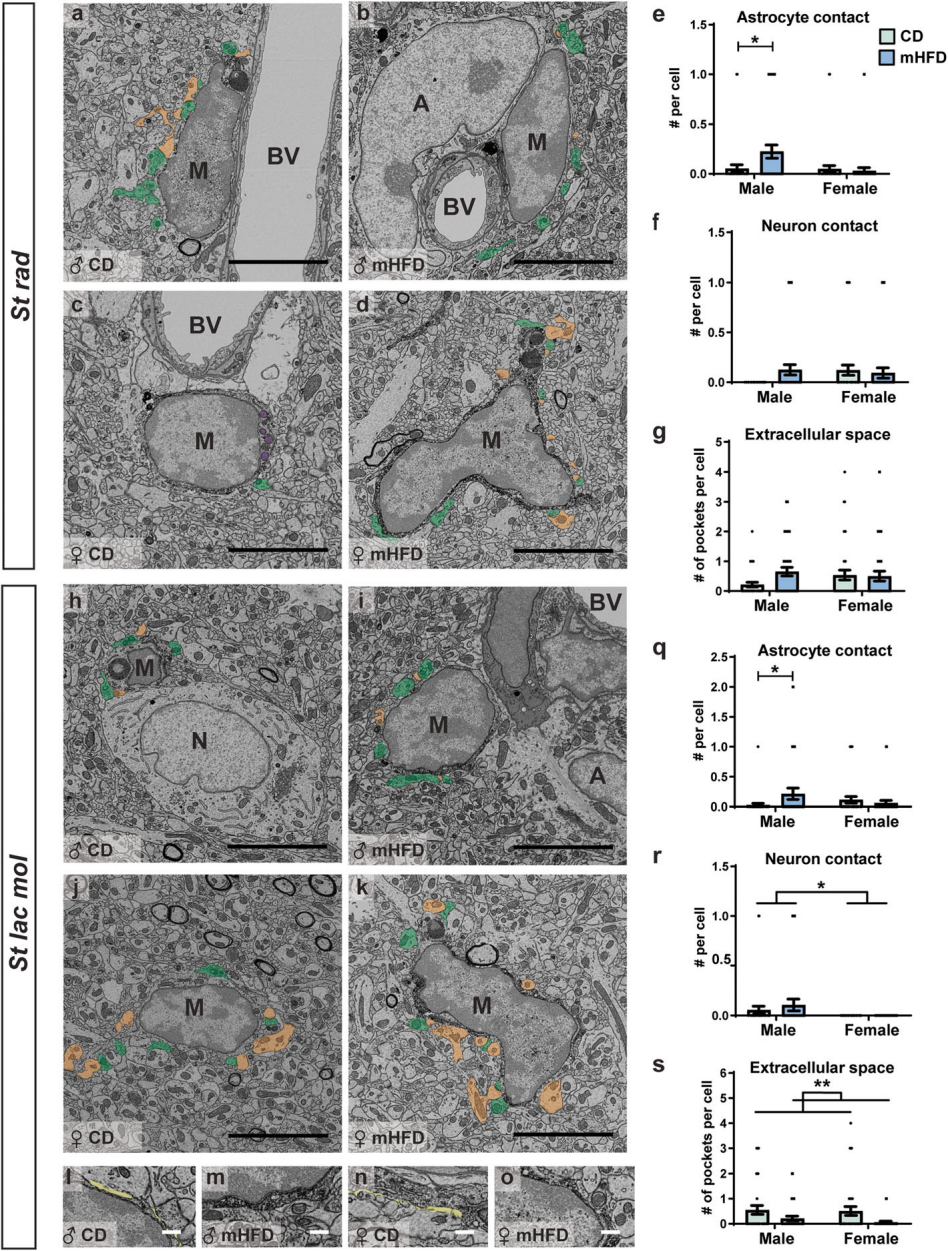
mHFD effect on microglial ultrastructure in the dorsal hippocampus CA1 of PND30 offspring. Ultrastructural analysis of microglia was performed in the **a**–**g** *st rad* and **h**–**s** *st lac mol*. **a**–**d**, **h**–**k** Representative pictures of microglia as well as **l**–**o** higher magnification views of extracellular space pockets are provided. The interactions of microglia with their microenvironment differed between groups; here, we present main results for microglial contacts with **e**, **q** astrocytes; **f**, **r** neurons; and **g**, **s** extracellular space pockets. On the representative pictures, astrocytes, blood vessels, microglia, and neurons are respectively identified by an “A,” by “BV,” by an “M,” and by an “N.” Presynaptic and postsynaptic elements are pseudo-colored respectively in green and orange. Mitochondria are pseudo-colored in purple and extracellular space pockets in yellow. Data are shown as mean ± standard error of the mean. *p <* 0.05* (sex) by 2-way ANOVA, *p <* 0.05^#^ (sex × diet) by 2-way ANOVA followed by Bonferroni post-hoc test. ♀, female; ♂, male; CD, control; mHFD, maternal high-fat diet

In the *st rad*, no significant difference in microglial organelles was observed between offspring groups, but there was a trend for a main diet effect regarding the total number of mitochondria per microglial cell body (*F*_(1,146)_ = 3.870, *p* = 0.0511) (Table 6). In terms of microglial interactions with their microenvironment, we detected a sex × diet interaction for the number of microglial contacts with astrocytes (*F*_(1,146)_ = 0.0446). Post-hoc analysis revealed that microglial cell bodies from mHFD-exposed male offspring made more cell-cell contacts with astrocytic cell bodies compared to CD male offspring (*p* = 0.0182, 0.225 ± 0.067 contacts vs 0.054 ± 0.038 contacts) (Table 6; Fig. 6a–e). Microglial interactions with synaptic elements, myelinated axons, neurons as well as oligodendrocytes remained unchanged (Table 6). We also identified a sex × diet interaction for the prevalence of microglia-associated extracellular digestion (*F*_(1,146)_ = 0.0433); however, post-hoc analysis revealed no significant different between offspring groups (Table 6).

**Table 6:** mHFD effects on microglial ultrastructure in the dorsal hippocampus CA1 *stratum radiatum* of PND30 offspring

Parameters			Mean ± standard error of the mean				***F***	***p***
			Male		Female			
			CD	mHFD	CD	mHFD		
Organelles	# Lysosome	Primary	1.405 ± 0.461	1.150 ± 0.317	0.878 ± 0.213	1.438 ± 0.258	Sex × diet, 1.552 Sex, 0.1344 Diet, 0.2160	Sex × diet, 0.2149 Sex, 0.7144 Diet, 0.6428
		Secondary	0.108 ± 0.052	0.200 ± 0.096	0.146 ± 0.075	0.188 ± 0.083	Sex × diet, 0.1028 Sex, 0.02644 Diet, 0.7068	Sex × diet, 0.7490 Sex, 0.8711 Diet, 0.4019
		Tertiary	0.027 ± 0.027	0.025 ± 0.025	0.000 ± 0.000	0.031 ± 0.031	Sex × diet, 0.5148 Sex, 0.2007 Diet, 0.3970	Sex × diet, 0.4742 Sex, 0.6548 Diet, 0.5296
	# Lipofuscin		0.135 ± 0.079	0.100 ± 0.048	0.073 ± 0.041	0.063 ± 0.043	Sex × diet, 0.04895 Sex, 0.8091 Diet, 0.1716	Sex × diet, 0.8252 Sex, 0.3699 Diet, 0.6793
	# Endosome	Empty	0.108 ± 0.052	0.200 ± 0.089	0.098 ± 0.058	0.063 ± 0.043	Sex × diet, 0.9319 Sex, 1.267 Diet, 0.1867	Sex × diet, 0.3360 Sex, 0.2621 Diet, 0.6663
		Content	0.432 ± 0.120	0.250 ± 0.128	0.268 ± 0.086	0.188 ± 0.070	Sex × diet, 0.2278 Sex, 1.132 Diet, 1.528	Sex × diet, 0.6339 Sex, 0.2890 Diet, 0.2185
	# Dilated ER/Golgi		5.892 ± 0.945	6.000 ± 1.046	6.171 ± 1.069	5.188 ± 0.723	Sex × diet, Sex, Diet,	Sex × diet, 0.5814 Sex, 0.7874 Diet, 0.6584
	# Mitochondrion	Elongated	0.459 ± 0.148	0.450 ± 0.138	0.293 ± 0.094	0.375 ± 0.087	Sex × diet, 0.1409 Sex, 0.9777 Diet, 0.08878	Sex × diet, 0.7080 Sex, 0.3244 Diet, 0.7662
		Total	2.270 ± 0.365	3.075 ± 0.486	2.195 ± 0.309	2.906 ± 0.322	Sex × diet, 0.01475 Sex, 0.1002 Diet, 3.870	Sex × diet, 0.9035 Sex, 0.7521 Diet, 0.0511
Interactions with microenvironment	# Synaptic element	Pre-synaptic	6.243 ± 0.504	9.075 ± 0.958	7.683 ± 0.808	7.688 ± 0.647	Sex × diet, 3.521 Sex, 0.008597 Diet, 3.111	Sex × diet, 0.0701 Sex, 0.9732 Diet, 0.0692
		Post-synaptic	3.081 ± 0.286	4.150 ± 0.438	3.707 ± 0.457	3.875 ± 0.559	Sex × diet, 1.028 Sex, 0.1562 Diet, 1.936	Sex × diet, 0.3123 Sex, 0.6933 Diet, 0.1663
	# Myelinated axon		0.135 ± 0.069	0.200 ± 0.089	0.073 ± .054	0.031 ± 0.031	Sex × diet, 0.6218 Sex, 2.949 Diet, 0.02917	Sex × diet, 0.4280 Sex, 0.0880 Diet, 0.8646
	# Degenerating myelin		0.216 ± 0.079	0.150 ± 0.067	0.098 ± 0.047	0.042 ± 0.042	Sex × diet, 0.006245 Sex, 3.020 Diet, 0.8740	Sex × diet, 0.9371 Sex, 0.0845 Diet, 0.3515
	# Brain cell or vasculature	Astrocyte	0.054 ± 0.038	0.225 ± 0.067	0.049 ± 0.034	0.031 ± 0.031	Sex × diet, 4.104 Sex, 4.576 Diet, 2.719	Sex × diet, 0.0446* Sex, 0.0341* Diet, 0.1013
		Neuron	0.000 ± 0.000	0.125 ± 0.053	0.122 ± 0.052	0.094 ± 0.052	Sex × diet, 2.771 Sex, 0.9714 Diet, 1.106	Sex × diet, 0.0981 Sex, 0.3260 Diet, 0.2946
		Oligodendrocyte	0.000 ± 0.000	0.000 ± 0.000	0.000 ± 0.000	0.000 ± 0.000	N/A	N/A
		Blood vessel	0.189 ± 0.065	0.075 ± 0.042	0.073 ± 0.041	0.031 ± 0.031	Sex × diet, 0.5805 Sex, 2.837 Diet, 2.709	Sex × diet, 0.4473 Sex, 0.0942 Diet, 0.1019
	# Extracellular space		0.216 ± 0.079	0.650 ± 0.146	0.537 ± 0.168	0.500 ± 0.168	Sex × diet, 2.596 Sex, 0.3406 Diet, 1.851	Sex × diet, 0.1093 Sex, 0.5604 Diet, 0.1757
	# Extracellular digestion		0.135 ± 0.057	0.275 ± 0.101	0.366 ± 0.120	0.125 ± 0.059	Sex × diet, 4.154 Sex, 0.1867 Diet, 0.2923	Sex × diet, 0.0433* Sex, 0.6663 Diet, 0.5896

*#* number, *CD* control diet, *ER/Golgi* endoplasmic reticulum and Golgi apparatus cisterna, *mHFD* maternal high-fat diet, *N/A* not applicable, *P*-values of significant statistical tests are in bold and followed by an asterisk indicating the significativity, * < 0.05

In *st lac mol*, microglial organelle content and ultrastructure were unaffected by offspring groups (Table 7), but their interactions with the microenvironment differed. Similar to microglia in the *st rad*, microglial cell bodies in *st lac mol* of mHFD-exposed male offspring had increased interactions with astrocytic cell bodies (*F*_(1,128)_ = 4.604, *p* = 0.0446) compared to CD male offspring (0.028 ± 0.028 contacts vs 0.214 ± 0.094 contacts) (Table 7; Fig. 6a–e, h–k, q). Of note, a significant main sex effect was also detected for microglial interactions with neurons (*F*_(1,128)_ = 6.062, *p* = 0.0151), where microglial cell bodies from male offspring compared to female offspring made more cell-cell contacts with neuronal cell bodies, regardless of maternal diet (0.081 ± 0.026 contacts vs 0.000 ± 0.000 contacts) (Table 7; Fig. 6h–k, r). Microglial interactions with synaptic elements, oligodendrocytes, and myelinated axons were also unchanged across sex and diet groups (Table 7). Lastly, mHFD-exposed offspring had a significant decrease (*F*_(1,128)_ = 7.666, *p* = 0.0065) in microglia-associated extracellular space pockets compared to CD offspring (0.1374 ± 0.0768 contacts vs 0.5349 ± 0.0206 contacts) (Table 7; Fig. 6l–o, s) in the two sexes.

**Table 7:** mHFD effects on microglial ultrastructure in the dorsal hippocampus CA1 *stratum lacunosum moleculare* of PND30 offspring

Parameters			Mean ± standard error of the mean				***F***	***p***
			Male		Female			
			CD	mHFD	CD	mHFD		
Organelles	# Lysosome	Primary	1.556 ± 0.419	1.357 ± 0.258	1.229 ± 0.239	1.182 ± 0.202	Sex × diet, 0.06292 Sex, 0.6902 Diet, 0.1644	Sex × diet, 0.8023 Sex, 0.4076 Diet, 0.6858
		Secondary	0.361 ± 0.090	0.250 ± 0.098	0.229 ± ± 0.092	0.212 ± 0.104	Sex × diet, 0.2389 Sex, 0.7742 Diet, 0.4337	Sex × diet, 0.6259 Sex, 0.3806 Diet, 0.5113
		Tertiary	0.028 ± 0.028	0.000 ± 0.000	0.000 ± 0.000	0.030 ± 0.030	Sex × diet, 1.817 Sex, 0.003435 Diet, 0.003435	Sex × diet, 0.1800 Sex, 0.9534 Diet, 0.9534
	# Lipofuscin		0.111 ± 0.053	0.107 ± 0.060	0.029 ± 0.029	0.061 ± 0.042	Sex × diet, 0.1493 Sex, 1.918 Diet, 0.09070	Sex × diet, 0.6999 Sex, 0.1684 Diet, 0.7638
	# Endosome	Empty	0.194 ± 0.078	0.107 ± 0.079	0.229 ± 0.101	0.091 ± 0.051	Sex × diet, 0.09748 Sex, 0.01231 Diet, 1.945	Sex × diet, 0.7554 Sex, 0.9118 Diet, 0.1655
		Content	0.361 ± 0.144	0.214 ± 0.079	0.171 ± 0.077	0.152 ± 0.063	Sex × diet, 0.3989 Sex, 1.579 Diet, 0.6886	Sex × diet, 0.5288 Sex, 0.2113 Diet, 0.4082
	# Dilated ER/Golgi		5.611 ± 0.788	7.643 ± ± 1.413	5.800 ± 0.805	7.512 ± 1.168	Sex × diet, 0.1075 Sex, 0.02125 Diet, 2.659	Sex × diet, 0.7436 Sex, 0.8843 Diet, 0.1054
	# Mitochondrion	Elongated	0.472 ± 0.180	0.500 ± 0.159	0.257 ± 0.118	0.485 ± 0.235	Sex × diet, 0.3099 Sex, 0.4110 Diet, 0.5061	Sex × diet, 0.5787 Sex, 0.5226 Diet, 0.4781
		Total	3.111 ± 0.534	2.071 ± 0.430	2.400 ± 0.341	2.970 ± 0.543	Sex × diet, 2.856 Sex, 0.03862 Diet, 0.2435	Sex × diet, 0.0935 Sex, 0.8445 Diet, 0.6225
Interactions with microenvironment	# Synaptic element	Pre-synaptic	6.250 ± 0.745	5.821 ± 0.587	5.171 ± 0.527	5.125 ± 0.442	Sex × diet, 0.1168 Sex, 2.138 Diet, 0.1406	Sex × diet, 0.7331 Sex, 0.1462 Diet, 0.7083
		Post-synaptic	3.306 ± 0.378	3.036 ± 0.369	3.629 ± 0.482	3.030 ± 0.300	Sex × diet, 0.1726 Sex, 0.1614 Diet, 1.206	Sex × diet, 0.6785 Sex, 0.6885 Diet, 0.2742
	# Myelinated axon		0.417 ± 0.108	0.536 ± 0.167	0.486 ± 0.161	0.545 ± 0.151	Sex × diet, 0.04059 Sex, 0.07164 Diet, 0.3689	Sex × diet, 0.8406 Sex, 0.7894 Diet, 0.5447
	# Degenerating myelin		0.083 ± 0.047	0.179 ± 0.090	0.200 ± 0.069	0.121 ± 0.058	Sex × diet, 1.763 Sex, 0.2047 Diet, 0.01575	Sex × diet, 0.1867 Sex, 0.6517 Diet, 0.9003
	# Brain cell or vasculature	Astrocyte	0.028 ± 0.028	0.214 ± 0.094	0.114 ± 0.055	0.061 ± 0.042	Sex × diet, 4.604 Sex, 0.3601 Diet, 1.408	Sex × diet, 0.0338* Sex, 0.5495 Diet, 0.2376
		Neuron	0.056 ± 0.039	0.107 ± 0.060	0.000 ± 0.000	0.000 ± 0.000	Sex × diet, 0.6095 Sex, 6.062 Diet, 0.6095	Sex × diet, 0.4364 Sex, 0.0151* Diet, 0.4364
		Oligodendrocyte	0.000 ± 0.000	0.036 ± 0.036	0.000 ± 0.000	0.000 ± 0.000	Sex × diet, 1.384 Sex, 1.384 Diet, 1.384	Sex × diet, 0.2417 Sex, 0.2417 Diet, 0.2417
		Blood vessel	0.056 ± 0.039	0.143 ± 0.067	0.114 ± 0.055	0.152 ± 0.063	Sex × diet, 0.2002 Sex, 0.3625 Diet, 1.238	Sex × diet, 0.6553 Sex, 0.5482 Diet, 0.2679
	# Extracellular space		0.556 ± 0.176	0.214 ± 0.094	0.514 ± 0.180	0.061 ± 0.042	Sex × diet, 0.1533 Sex, 0.4611 Diet, 7.666	Sex × diet, 0.6961 Sex, 0.4984 Diet, 0.0065**
	# Extracellular digestion		0.528 ± 0.216	0.321 ± 0.219	0.200 ± 0.090	0.364 ± 0.105	Sex × diet, 1.231 Sex, 0.7334 Diet, 0.01641	Sex × diet, 0.2693 Sex, 0.3934 Diet, 0.8983

*#* number, *CD* control diet, * ER/Golgi* endoplasmic reticulum and Golgi apparatus cisterna, *mHFD* maternal high-fat diet, *N/A* not applicable, *P*-value of significant statistical tests are in bold and followed by the number of asterisks indicating the significativity, * < 0.05, ** < 0.01

### Dark microglia and perivascular cells display increased number of dilated endoplasmic reticulum and Golgi apparatus cisterna in mHFD offspring

**Fig. 7:**
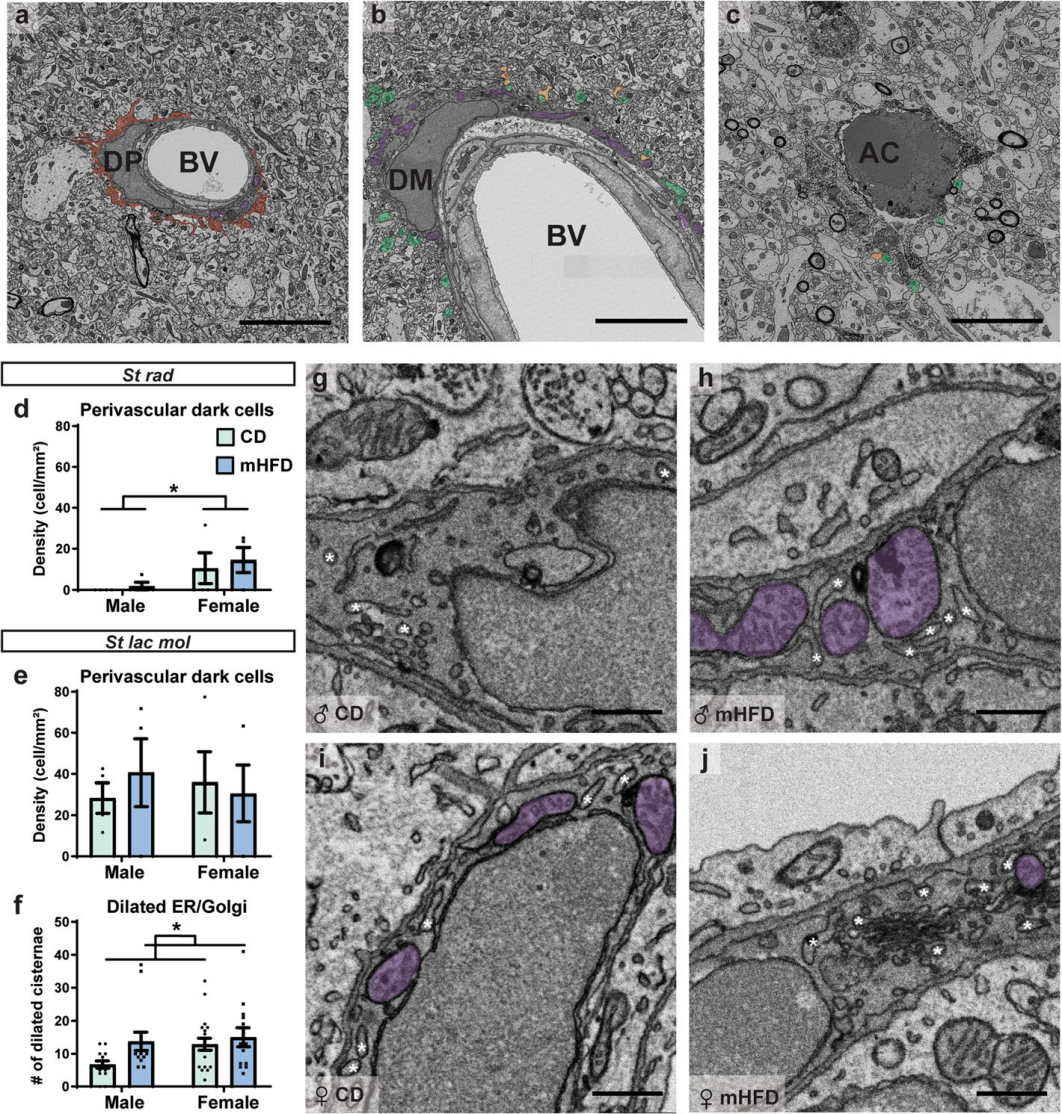
mHFD effect on dark perivascular cells, dark microglial cells, and apoptotic cells in dorsal hippocampus CA1 of PND30 offspring. **a** Dark perivascular cells, **b** dark microglia, and **c** apoptotic cells examples are presented. Dark perivascular cell density was counted in the **d** *st rad* and the **e** *st lac mol*. Ultrastructural analysis of dark cells in the *st lac mol* revealed differences in their **f**–**j** dilation of the endoplasmic reticulum and Golgi apparatus cisternae. Dark processes are pseudo-colored in red. Presynaptic and postsynaptic elements are pseudo-colored respectively in green and orange. Mitochondria are pseudo-colored in purple. Blood vessels are identified by “BV.” Dark perivascular cell, dark microglia, and apoptotic cell are respectively identified using “DP,” “DM,” and “AC.” Dilated endoplasmic reticulum and Golgi apparatus are identified by a white asterisk. Scale bar for representative picture of cell type is equivalent to 5 μm while scale bar for picture showing endoplasmic reticulum and Golgi apparatus is equivalent to 500 nm. Data are shown as mean ± standard error of the mean. *p <* 0.05* (sex) by 2-way ANOVA, *p <* 0.05^#^ (sex × diet) by 2-way ANOVA followed by Bonferroni post-hoc test. ♀, female; ♂, male; CD, control; Dilated ER/Golgi, dilated endoplasmic reticulum and Golgi apparatus cisternae; mHFD, maternal high-fat diet

Previously, our laboratory identified a microglial subset, the “dark microglia,” which is characterized by a distinct ultrastructural signature compared with typical microglia. These cells are found within the brain parenchyma, notably in the ventral/dorsal hippocampus CA1 *st rad* and *st lac mol*. Dark microglia exhibit several markers of cellular stress (dilatation of endoplasmic reticulum and Golgi, elongated mitochondria) as well as a dark, electron-dense cytoplasm, and nucleoplasm [69]. These stressed microglia are rare in healthy mature mice but become abundant in pathological conditions [69] including in a MIA mouse model induced with polyinosinic polycytidylic acid (polyinosinic–polycytidylic acid) [54]. In the current study, we characterized the density and ultrastructure of dark microglia in the dorsal hippocampus CA1, *st rad* and *st lac mol*, comparing mHFD with CD offspring at PND30. While imaging, we also noticed intriguing dark perivascular cells, localized inside the perivascular space yet displaying dark features similar to the dark microglia (i.e., dark, electron-dense cytoplasm, and nucleoplasm, as well as markers of cellular stress). We further encountered apoptotic cells, identified by their dark cytoplasm, which was accompanied in this case by a distinctive pyknotic and fragmented nucleus. We decided to also quantify their density. The quantitative analysis of dark microglia and apoptotic cells revealed no significant difference in their density among the *st rad* and *st lac mol* of the adolescent offspring, regardless of their sex and maternal diet (Supplementary Table 1; Fig. 7a–c). Notwithstanding, more than half of the apoptotic cells we observed (four out of seven) were identified as microglia by their IBA1^+^ staining. In the *st rad*, dark perivascular cells also displayed a similar density between offspring groups. In the *st lac mol*, however, a sex difference was observed, with the female offspring showing a significantly increased density of dark perivascular cells (*F*_(1,12)_ = 5.692, *p* = 0.0344) compared to male offspring (12.58 ± 1.99 cell/mm^2^ vs 0.94 ± 0.94 cell/mm^2^) (Supplementary Table 1; Fig. 7d–e); regardless of maternal diet.

To study changes in organelles among the stressed dark cells in the *st lac mol*, we pooled together dark microglia and dark perivascular cells to obtain a sufficient sample size, required to be around 50 individual cells total for a large effect size (~ 0.4) (also see Supplementary Table 2 for the semi-descriptive analysis of the dark microglia and dark perivascular cells considered separately). This quantitative analysis of dark cells revealed a main diet effect on their number of dilated endoplasmic reticulum or Golgi apparatus cisternae, which significantly increased (*F*_(1,55)_ = 4.264, *p* = 0.0437) in mHFD compared to CD offspring (14.38 ± 0.62 dilated cisterna vs 9.805 ± 3.090 dilated cisterna) (Supplementary Table 3; Fig. 7f–j). In addition, secondary lysosomes were significantly more abundant in female offspring compared to male offspring regardless of their maternal diet (female offspring 0.453 ± 0.120 lysosomes vs male offspring 0.077 ± 0.109 lysosomes) (Supplementary Table 3). This finding may describe a sex difference, regardless of maternal diet, in terms of dark cells phagolysosomal pathways. Across groups, the two types of stressed dark cells lastly displayed in the *st lac mol* similar numbers of lysosomes, lipofuscin, endosomes, and mitochondria, and their relationships with the microenvironment did not differ between groups.

## Discussion

Our study investigated the effects of a fat enriched maternal diet on peripheral immune and microglial properties in the adolescent offspring of each sex. Characterization of our mouse model revealed a MIA phenotype defined by elevated circulating IL-6 in the mothers, together with increased fat deposition in the mothers and male offspring without other major metabolic changes. This MIA model led to peripheral immune priming demonstrated by exacerbated release of IL-6 upon an LPS-induced immune challenge in mHFD-exposed male and female offspring. The mHFD-induced MIA resulted in significant microglial morphological changes in the dorsal hippocampus CA1. We also found sexually dimorphic hippocampal transcriptomic changes, with mHFD-exposed male offspring showing reduced mRNA expression of the inflammatory-regulating mediators *Nfκb* and *Tgf1b*, and microglial receptors *Tmem119*, *Trem2*, and *Cx3cr1*. In parallel to these changes, mHFD-exposed male offspring had increased microglial interactions with astrocytes in the dorsal hippocampus CA1, while both mHFD males and females had decreased microglia-associated extracellular space pockets in the same region. Taken together, this data highlights the emergence of a partially sex-dependent priming that lasted until adolescence in the mHFD offspring.

Previous works studying the metabolic consequences of mHFD in rodents have reported variable effects. With the same diet protocol that we used but in rats, Sasaki et al. observed that weight differences varied with the age of the offspring across their lifespan upon exposure to mHFD [13, 32]. mHFD offspring were significantly heavier during pubertal stages (PND8 to PND21) [13], while at adolescence (PND35) [32] and adulthood (PND90), their weight was similar to control animals [13]. Similarly, we did not find any significant difference of body weight in the PND30 adolescent mouse offspring. By contrast, during early postnatal ages (PND1 to PND10), rat offspring exposed to lower fat mHFD (43% kcal/fat vs our 60% kcal/fat) became heavier with an increased fat mass [89], similar to our increased fat mass of mHFD-exposed male offspring. However, our P30 mHFD offspring showed no change in body weight that could be compensated notably by loss or change in composition of bone density, which has been previously observed after exposure to mHFD [90–92]. Future investigation using bone densitometry analysis could determine if that is also the case in our mHFD mouse model. The study also reported heavier dams with lower blood glucose levels prior to mating [89], describing a diet-induced obesity model. Bilbo and Tsang compared two types of mHFD in rats—one with saturated fat and the other with trans-fat—with the same protocol duration used here and found that both diets led to heavier dams [8]. In offspring, exposure to mHFD rich in saturated fats, and not trans-fats, led to significant weight grain in both sexes at puberty (PND20) and in males at adulthood (PND60) [8]. Also in rats, maternal programming after a HFD increased offspring body weight later in adulthood (> 6 weeks of age), whereas offspring exposed to a maternal high-sugar-fat diet were transiently lighter in early adulthood (4–5 weeks of age) [93]. In this latter study, dams fed with a HFD (45% kcal/fat) did not present any weight gain, while dams fed with high-sugar-fat diet weighed lower during gestation [93]. Overall, these studies suggest that the diet composition and exposure time may differently impact on the dams and offspring metabolism and that there might be slight differences between species (e.g., mouse vs rat). In comparison, our mHFD mouse model did not result in severe endocrinological alterations (i.e., obese or diabetic-like phenotype) in the dams, suggesting that the effects observed in our offspring were primarily due to the immunologic consequences of a fat enriched diet during pregnancy and nurturing, without confounding effects of obesity-induced metabolic dysregulation.

Few studies have examined the MIA induced by mHFD as well as the mechanisms underlying these MIA effects of mHFD. Using a macaque model of mHFD, Thompson et al. have noted an elevation of the pro-inflammatory cytokine IL-12 and a decrease of macrophage-derived chemokine (also known as C-C motif chemokine 22) in the blood of the mothers [9]. This study was the first to directly highlight the interplay among metabolic and inflammatory maternal changes resulting from the diet and their consequences on the offspring behaviors. In rodents, Bilbo and Tsang did not observe an increase in IL-6 in rat dams fed with a high saturated fat diet [8], contrary to our study. However, subsequent studies using a mouse model as well as a more sensitive approach involving multiplex-ELISA observed increased levels of circulating cytokines, including IL-6, in dams, during pregnancy [75] and the end of nurturing [14]. Discrepancy between Bilbo and Tsang with ours’ and others’ results may be explained by the difference in the sensitivity between the techniques. In addition, differences between the diets or species used could also underlie this discrepancy. Interestingly, pregnant female rat fed with a HFD had increased mRNA levels of *Il-6* in the placenta [75], suggesting that IL-6 could cross the placental barrier to modulate fetal development [94] in our model.

In the mHFD-exposed offspring, alterations of the inflammatory status have notably been highlighted by increased cytokines levels (i.e., IL-5) in the blood of male mice at PND7 and of female mice at PND21 [14]. Moreover, another independent study has reported increased peripheral IL-6 4 h after an LPS immune challenge in adult rat male and female offspring [8]. In agreement with our results, these data suggest that during both adolescence and adulthood, mHFD-exposed offspring have a stronger response to a systemic immune challenge. IL-6 could contribute to inducing immune programming changes in the offspring by modifying transcriptional regulation of inflammatory mediators that are part of IL-6 downstream signaling target (i.e., *AP1*, *NFκB*, *Sp1*) [76]. It is also possible that a stronger response to immune challenge occurs through increased release of cytokines by fat deposits. In the homeostatic central nervous system, Bilbo and Tsang reported an increase of protein levels of IL-1β without change of mRNA levels of *Il-1β* and *Il-6* in PND20 pubertal and PND60 adult offspring, from both sexes [8]. Another group, Sasaki et al., revealed greater mRNA levels of *Il-6* in the adolescent hippocampus [32] and no difference compared to control in the adult hippocampus [13] using a similar diet protocol in male and female rats. In the present study, we did not detect *Il-1 β* or *Il-6* mRNA, suggesting levels below threshold for detection by rt-qPCR or a lower efficiency of the primers used. Although Sasaki et al. focused on an adolescent timepoint, they looked later, at PND45, and in rats. Therefore, further investigations are necessary to appraise the adolescence immune changes in mHFD rodent models.

In parallel to the changes of pro-inflammatory genes, neuroendocrine regulator genes (i.e., *Gr*, *Mr*, *Nfκb)* were also reported to be differently expressed in the rat hippocampus across adolescence and adulthood [13, 32, 95]. These neuroendocrine receptors can modulate microglial functions such as their release of inflammatory mediators (i.e., IL-1β, IL-6, TNF-α) during an LPS immune challenge [96–98], their morphology [99], cellular dynamics, and proliferation [98]. Moreover, neuroendocrine receptors are differently expressed by microglia between the sexes [100]. In the current study, we found increased expression of *Cox2* and *Nfκb* 8 h after LPS; however, these genes were similarly increased regardless of the maternal diet. In homeostatic condition, we observed a sexual dimorphism of the transcription factor *Nfκb* and the homeostatic-regulating cytokine *Tgfb1*, with lower expression levels detected in whole hippocampus of mHFD-exposed male offspring compared to other offspring groups. Decrease of *Nfκb* in SAL-treated animal suggests changes in its homeostatic functions, encompassing homeostatic regulation of neuronal excitability that may contribute to behavioral impairments [77]. Moreover, decrease of *Tgfb1* expression indicates a loss of microglial homeostatic signature, which could affect their physiological functions and suggest a priming mechanism induced by mHFD. These transcriptional changes may be linked to aberrant behavioral outcomes. However, we cannot conclude from our present work on a direct relationship considering that the behavioral alterations were similar between sexes.

mHFD studies have mainly examined its effects on global inflammation and gene expression changes, without focusing on microglia. However, microglia may represent one of the key actors mediating the pathological consequences of mHFD during neurodevelopment. Bilbo and Tsang previously reported higher density of IBA1^+^ cells in the hippocampus CA1, CA3, and dentate gyrus of mHFD-exposed male and female rat offspring at adulthood [8]. Similar effect of IBA1^+^ increase has also been recently reported in the hypothalamus of rat offspring exposed to a maternal overnutrition model (high-sugar-fat) [45]. In the present study, we did not observe any change in microglial density in the *st rad* and *st lac mol* of the dorsal hippocampus CA1. This could be explained by the method of analysis where microglia are identified by integrated density of IBA1^+^ staining among hippocampal regions (CA1, CA3, dentate gyrus) vs count of IBA1^+^/TMEM119^+^ cells in individual CA1 layers, together with the age or the species of animals used. Nevertheless, we observed microglial morphological changes in the *st rad* (circularity) and in *st lac mol* (solidity), as well as a decreased average branch length in the *st lac mol*. The reduced microglial circularity in the *st rad* of mHFD-exposed offspring suggests that these cells took a more elongated shape. Microglia with an elongated, rod-like morphology have been previously proposed to play a role in the response to acute brain insults due to their increased prevalence [101]. Elongated bipolar microglia were also previously described in a viral MIA model, in which ex vivo analyses revealed that microglial chemotaxis and phagocytosis are increased in response to treatment (chemotaxis: CCL-2, IL-8, phagocytosis: LPS, poly I:C) [102]. The increased microglial solidity may represent a change in the distribution of the arborization, particularly a decreased distance between branches which would reflect a more convex-like shape. Others also hypothesized that an increase of the solidity value occurs during the morphological shift from a ramified to an amoeboid shape upon neuroinflammatory insults and represents a de-ramified or bushy morphology [103, 104], a phenotype seen upon exposure to stress [105]. In a bacterial MIA mouse model, microglia were previously shown to shift their morphology to an ameboid shape in adolescent (PND40) offspring amygdala [106]. Although the microglial morphological changes we observed varied between layers, similarly they could indicate a general shift from a surveillant-ramified state to an amoeboid shape. This morphological shift may also accompany microglial functional alterations in the hippocampus CA1 of mHFD offspring.

Microglia from the mHFD vs CD offspring interacted differently with their microenvironment. In *st lac mol*, the presence of extracellular space pockets surrounding microglial cell bodies decreased, which could be explained for instance by changes in the composition of the extracellular matrix [107]. Microglia themselves contribute to modifying the extracellular matrix by releasing cathepsins, heparinases, and metalloproteinases [108, 109] that promote their cell body migration and process motility, notably during inflammation [108, 109], as well as experience-dependent plasticity during normal physiological conditions [36, 110]. Hence, the reduction in microglia-associated extracellular space that we measured could indicate a decrease in microglial dynamics associated with impaired physiological functions.

Together with the decreased expression of microglial receptors (i.e., *Cx3cr1*, *Tmem119*, *Trem2*), the morphological and functional changes that we observed could be partially caused by the transcriptomic alterations in adolescent male offspring. Although its role remains under investigation, TMEM119 is constitutively and exclusively expressed by microglia [55] and is required for their survival [81]. However, the absence of changes in the microglial and infiltrating myeloid cells population size suggest no major issue with microglia survival, without excluding the possibility of a change in the turnover of the population. Similar to TMEM119, TREM2 aids microglia to survive, in addition to mediating physiological functions that include blood flow regulation [111], phagocytosis, and synaptic pruning [82, 85–87]. CX3CR1 is also involved in microglial phagocytosis and synaptic remodeling [83, 84, 112]. Hence, a decrease of *Cx3cr1* and *Trem2* mRNA expression may reflect reduced microglial interactions with neurons or synaptic elements as well as phagocytic activities, which was not observed at the ultrastructural level. It should be noted however that our ultrastructural analysis focused on microglial cell bodies, leaving the possibility that microglial processes make less contacts with synapses, which remains to be explored. A recent study suggested that microglial TREM2 may be involved in the mediation of microglia-astrocyte crosstalk. Using a TREM2 knockout mouse model, TREM2 deficiency led to a lack of microglia-astrocyte interactions in the cerebral cortex and hippocampus, which prevented the astrocytic-mediated phagocytosis of axon terminals [113]. The decrease of TREM2 that we observed may represent a partial loss of microglial control over this newly defined function of astrocytes at synapses. Taken with the ultrastructural finding of male-specific increase of microglial interactions with astrocytes, these results stress the importance of determining the molecular crosstalk between microglia and astrocytes that is at play in mHFD-exposed male offspring. Interestingly, investigating epigenetic changes of the oxytocin receptor in the hippocampus of mHFD-exposed male and female mouse offspring by chromatin immunoprecipitation-qPCR revealed a sexually dimorphic pattern of acetylation and methylation leading to an increased expression of the oxytocin receptor in the hippocampus of male offspring only [114]. Further investigation looking at whole genome epigenomic changes could hence provide, in parallel with post-translational modifications and/or protein expression analysis, better understanding of how microglial function differs between sexes upon mHFD.

Our laboratory has previously identified dark microglia as a subset of microglia associated with pathological conditions that are characterized by a dark cytoplasm and nucleoplasm without a clear chromatin pattern [54, 69]. Not only microglia can undertake a “dark” appearance but several other types of brain cells such as neurons, oligodendrocytes [58, 66], and astrocytes [115, 116] as well. Here, we not only observed dark microglia but also stressed dark perivascular cells showing several signs of cellular stress (i.e., elongated mitochondria and dilated cisternae of the endoplasmic reticulum and Golgi apparatus) and dying apoptotic cells sometimes immunopositive for IBA1 in the dorsal hippocampus CA1 of the PND30 offspring. No difference in the density of stressed dark cells and apoptotic cells was observed with the maternal diet. However, an interesting observation we made was the increased density of stressed dark perivascular cells in the *st rad* of female offspring compared to male offspring from both maternal diets. Unfortunately, the low sample size of stressed dark cells and apoptotic cells did not allow us to perform quantitative analysis in the dorsal hippocampus CA1 *st rad*. Similar to previous observations by our team in adult pathological conditions, dark microglia were mainly found to be located in the *st lac mol* among the CA1 [69]. We thus assessed the global changes in stressed dark cells organelles without discriminating dark microglia from dark perivascular cells to increase our sample size. This analysis of dark microglia and dark perivascular cells revealed a significantly increased number of dilated endoplasmic reticulum and Golgi apparatus cisternae. Dilation of the endoplasmic reticulum and the Golgi apparatus are well characterized ultrastructural signs of oxidative stress [117]. The increase in their incidence thus reflects a higher level of cellular stress in the dorsal hippocampus CA1 of mHFD-exposed adolescent offspring. It would be interesting to investigate the cellular stress signature of the two cell types at the molecular level and the effects of dark microglia as well as dark perivascular cells on the blood-brain barrier. In fact, increased stress within the neurovascular unit could alter blood-brain barrier integrity and permeability, as well as the regulation of blood flow. Consequently, a dysfunctional blood-brain barrier may leak into the brain peripheral inflammatory signals released upon mHFD, such as IL-6, thus contributing to the microglial changes observed.

## Conclusion

Although mHFD induced similar phenotypes in both sexes for IL-6-driven immune priming and microglial morphology, we identified sex-specific effects in the mHFD male offspring in terms of transcriptomic (*Nfκb*, *Tgf1b*, *Tmem119*, *Trem2*, *Cx3cr1*) as well as ultrastructural (astrocyte-microglia interaction) changes during adolescence. Considering their key role in shaping brain neuronal networks, microglia in dorsal hippocampus may be partially responsible for several pathological neurodevelopmental outcomes described in mHFD models. Adolescence is a period of intense brain plasticity and maturation, where several microglia-mediated processes, such as synaptic pruning and myelination, are ongoing and may be profoundly impacted by the mHFD-driven microglial priming. Notably, synaptic pruning may be especially impacted negatively in mHFD-exposed male offspring. Investigation into these specific neurodevelopmental processes across the brain and determining the kinetics of their alterations during the offspring neurodevelopment, from embryonic stages to young adulthood, will be central to understanding the sexually dimorphic pathological cascade involving microglia as well as microglia-astrocyte crosstalk.

## Supplementary information

**Additional file 1:.** Supplementary methods [118]

**Additional file 2: Supplementary Figure 1**. Weight, weight gain, food, and calories follow-up throughout diet protocol on the dams.

**Additional file 3: Supplementary Figure 2**. HFD effect on blood glucose, fat deposition, gestation duration and litter size.

**Additional file 4: Supplementary Figure 3**. mHFD effect on fat deposition in the PND30 offspring.

**Additional file 5: Supplementary Table 1**. mHFD effects on the density of dark microglia, dark perivascular cells, and apoptotic cells in the dorsal hippocampus CA1 *stratum radiatum* and *stratum lacunosum moleculare* of PND30 offspring.

**Additional file 6: Supplementary Table 2**. mHFD effects on the ultrastructure of dark cells in the dorsal hippocampus CA1 *stratum radiatum* of PND30 offspring.

**Additional file 7: Supplementary Table 3**. mHFD effects on the ultrastructure of dark cells in the *stratum lacunosum moleculare* of the dorsal hippocampus CA1 of PND30 offspring.

## Data Availability

The data that support the findings of this study are available from the corresponding author, MET, upon reasonable request.

## Abbreviations

AP-1: Activator protein-1
Aif1: Allograft inflammatory factor 1
a.u.: Arbitrary units
ANOVA: Analysis of variance
CA: Cornu ammonis
CD: Control chow
cDNA: Complementary deoxyribonucleic acid
Cox2: Cyclooxygenase 2
Ct: Cycle threshold
x3cr1: Fractalkine receptor
DAB: Diaminobenzidine
ED: Embryonic day
ELISA: Enzyme-linked immunosorbent assay
Gapdh: Glyceraldehyde 3-phosphate dehydrogenase
HFD: High-fat diet
IBA1: Ionized calcium-binding adapter 1
IL: Interleukin
Il1ra: IL1 receptor antagonist
IκB: Nfκb inhibitor
LPS: Lipopolysaccharide
mHFD: Maternal HFD
MIA: Maternal immune activation
MKP-1: Mitogen-activated kinase protein 1
mRNA: Messenger RNA
Nfκb: Nuclear factor κ-light-chain-enhancer activator of B cells
PND: Postnatal day
PB: Phosphate buffer
PBS: Phosphate buffered saline
PFA: Paraformaldehyde
RNA: Ribonucleic acid
RPL32: Ribosomal protein L32
rt-qPCR: Real-time quantitative polymerase chain reaction
SAL: Saline
SEM: Scanning electron microscopy
Sp-1: Specificity protein 1
*St lac mol*: *Stratum lacunosum moleculare*
*St rad*: *Stratum radiatum*
TBS: Tris buffered saline
Tgf1β R: Transforming growth factor beta 1 receptor
TMEM119: Transmembrane protein 119
TNF: Tumor necrosis factor
Trem2: Triggering receptor expressed on myeloid cells 2
W: Week
