# Supplementary material for: Microglial and peripheral immune priming is partially sexually dimorphic in adolescent mouse offspring exposed to maternal high-fat diet
Source: J Neuroinflammation. 2020 Sep 5;17:264. doi: 10.1186/s12974-020-01914-1 (PMC7487673; doi:10.1186/s12974-020-01914-1)
Supplement: Supplementary file 1 — Additional file 1:. Supplementary methods [118] [file 12974_2020_1914_MOESM1_ESM.docx]

## Supplementary

### Supplementary methods

#### Gestation and litter

Four cohorts of females were used in this study. For each cohort, during 3-day mating period, plug check was performed every morning to assess gestation (n=11-15 dams/diet). The day when a vaginal plug was observed was considered as embryonic day (ED)0.5. Between ED16-ED21, the cages were cautiously examined in the morning and evening to determine when females gave birth. The day at which movement in the nest or presence of newborn was observed was considered as the PND0 for the offspring. Of note, four CD dams and nine HFD dams cannibalised their litter early postpartum (data not shown). Duration of the gestation was determined by the time lapse between observation of vaginal plug and presence of the pups. Sexing of the offspring was performed at weaning of the litter (PND21). Of note, four of the 34 litters had only males or only females (n=17 litters/diet).

#### Weight follow-up

Dams (n=17 dams/diet) were weighed once a week during cage changes to limit stress induced by animal handling. Weight was averaged into following diet time points: start of the diet, mating, end of gestation, and end of nurturing. Weight gain was calculated after 4 weeks of diet consumption, and at the end of gestation and nurturing, by subtracting from the measured weight the weight of the preceding time period.

#### Food consumption follow-up

Throughout the diet protocol, the dams (n=17 dams/diet) had their food pellets weighed during the food changes every 3-4 days. The caloric and macronutrients (carbohydrate, fat and protein) consumption was calculated by multiplying the weight of food consumed by the caloric (Kcal/g) and macronutrients values of the diets (CD or HFD; Supplementary Figure 1 e).

#### Glucose random assay and fat deposits dissection

At the end of the diet protocol, nurturing dams (n=5-7 animals/diet) were weighed, and a blood sample was collected by submandibular puncture during the middle of the inactive phase (14:00). One drop of blood was used to measure circulating glucose blood levels using a OneTouch Ultramini blood glucometer (Johnson and Johnson, New Brunswick, NJ, United States). Blood glucose values higher than 250 mg/dL were considered as an indicator of hyperglycemia, as often seen in rodent models of diet-induced diabetes [118].

For fat distribution changes, animals –dams (n=6-7 animals/diet) and a cohort of PND30 offspring (n=5-6 animals/sex/diet)– were anesthetized with rodent cocktail (0.3 mL/100 g), weighed and decapitated. The bodies of the animals were frozen and kept at -20 °C until fat dissection. Brown, peritoneal, perigonadal and subcutaneous fat were dissected out and weighed (Supplementary Figure 2c). Weight of each fat deposits was reported on total body weight before sacrifice to assess body fat distribution.
