# Supplementary figures and images for: Microglial and peripheral immune priming is partially sexually dimorphic in adolescent mouse offspring exposed to maternal high-fat diet
Source: J Neuroinflammation. 2020 Sep 5;17:264. doi: 10.1186/s12974-020-01914-1 (PMC7487673; doi:10.1186/s12974-020-01914-1)

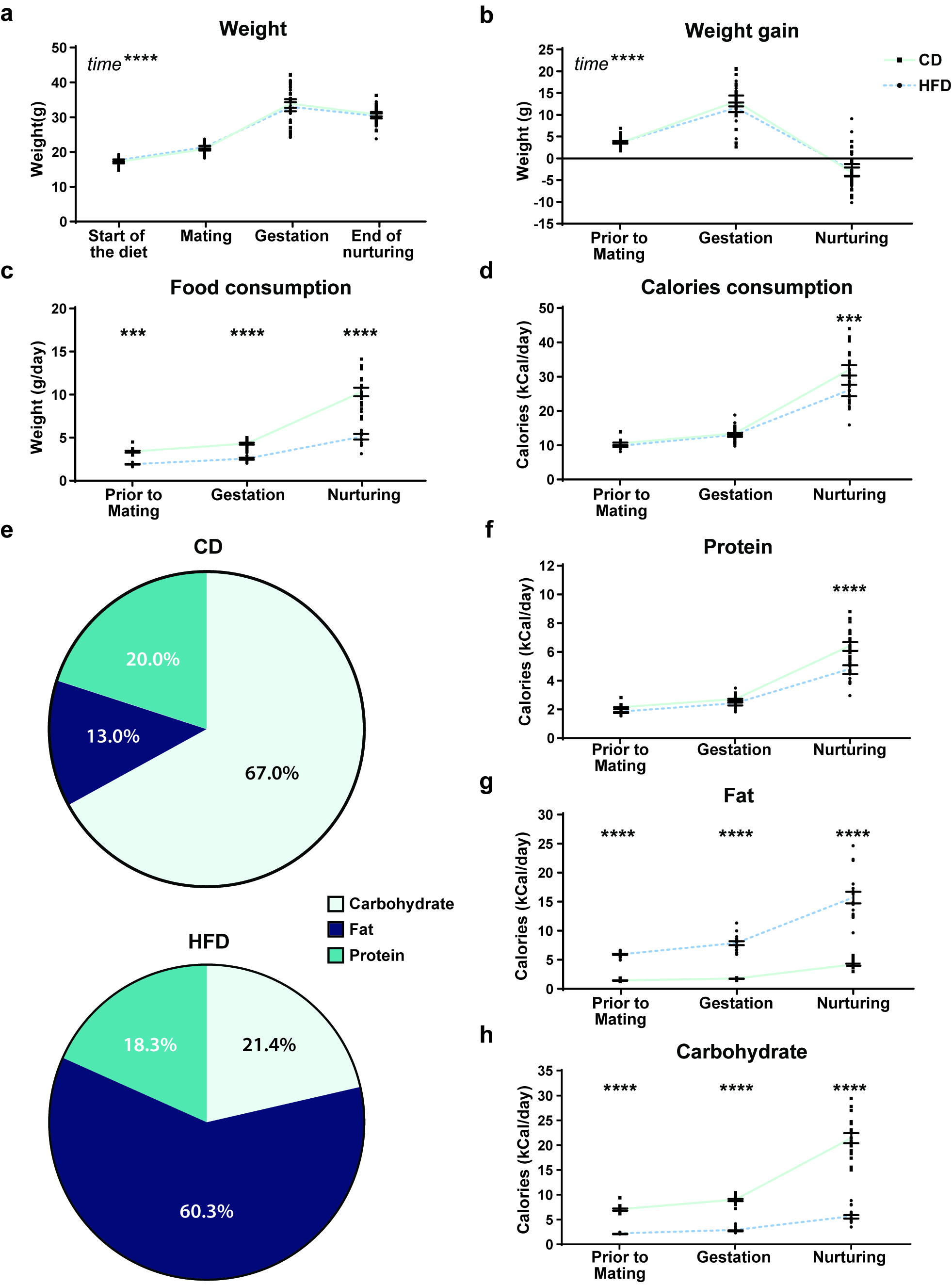

Supplement: Supplementary file 2 — Additional file 2: Supplementary Figure 1. Weight, weight gain, food, and calories follow-up throughout diet protocol on the dams. [file 12974_2020_1914_MOESM2_ESM.tif]
