# Supplementary material for: Microglial and peripheral immune priming is partially sexually dimorphic in adolescent mouse offspring exposed to maternal high-fat diet
Source: J Neuroinflammation. 2020 Sep 5;17:264. doi: 10.1186/s12974-020-01914-1 (PMC7487673; doi:10.1186/s12974-020-01914-1)
Supplement: Supplementary file 5 — Additional file 5: Supplementary Table 1. mHFD effects on the density of dark microglia, dark perivascular cells, and apoptotic cells in the dorsal hippocampus CA1 stratum radiatum and stratum lacunosum moleculare of PND30 offspring. [file 12974_2020_1914_MOESM5_ESM.docx]

**Supplementary Table 1. mHFD effects on the density of dark microglia, dark perivascular cells, and apoptotic cells in the dorsal hippocampus CA1 *stratum* *radiatum* and *stratum lacunosum moleculare* of PND30 offspring.** CD: control diet, *St lac mol*: *stratum* *lacunosum moleculare*, mHFD: maternal high-fat diet, *St rad*: *stratum radiatum, P-value* of significant statistical tests are in bold and followed by an asterisk indicating the significativity, * < 0.05.

| **Parameters** | | **Mean ± standard error of the mean** | | | | ***F*** | ***p*** |
| --- | --- | --- | --- | --- | --- | --- | --- |
|  |  | **Male** | | **Female** | |  |  |
|  |  | **CD** | **mHFD** | **CD** | **mHFD** |  |  |
| *St rad* | Density dark microglial cells (cell/mm²) | 1.581 ± 1.581 | 6.152 ± 3.726 | 2.064 ±1.297 | 0.000 ±0.000 | Sex*Diet: 2.437  Sex: 1.779  Diet: 0.3479 | Sex*Diet: 0.1444  Sex: 0.2071  Diet: 0.5662 |
|  | Density dark perivascular cells (cell/mm²) | 0.000 ± 0.000 | 1.883 ± 1.883 | 10.595 ±7.452 | 14.571 ± 6.014 | Sex*Diet: 0.04600  Sex: 5.692  Diet: 0.3604 | Sex*Diet: 0.8338  Sex: **0.0344***  Diet: 0.5595 |
|  | Density apoptotic cells (cell/mm²) | 0.999 ±0.999 | 0.000 ±0.000 | 0.000 ±0.000 | 0.000 ±0.000 | Sex*Diet: 1.000  Sex: 1.000  Diet: 1.000 | Sex*Diet: 0.3370  Sex: 0.3370  Diet: 0.3370 |
| *St lac mol* | Density dark microglial cells (cell/mm²) | 7.765 ± 5.765 | 5.288 ± 3.054 | 3.811 ± 2.418 | 3.781 ± 2.501 | Sex*Diet: 0.1103  Sex: 0.5493  Diet: 0.1158 | Sex*Diet: 0.7455  Sex: 0.4729  Diet: 0.7395 |
|  | Density dark perivascular cells (cell/mm²) | 28.41 ±7.40 | 40.70 ± 16.41 | 36.02 ± 14.80 | 30.61 ± 13.77 | Sex*Diet: 0.4267  Sex: 0.008405  Diet: 0.06455 | Sex*Diet: 0.5259  Sex: 0.9285  Diet: 0.8037 |
|  | Density apoptotic cells (cell/mm²) | 4.500 ± 2.898 | 2.353 ± 1.373 | 2.870 ± 1.921 | 0.000 ± 0.000 | Sex*Diet: 0.03744  Sex: 1.135  Diet: 1.802 | Sex*Diet: 0.8498  Sex: 0.3076  Diet: 0.2044 |
