# Supplementary material for: Microglial and peripheral immune priming is partially sexually dimorphic in adolescent mouse offspring exposed to maternal high-fat diet
Source: J Neuroinflammation. 2020 Sep 5;17:264. doi: 10.1186/s12974-020-01914-1 (PMC7487673; doi:10.1186/s12974-020-01914-1)
Supplement: Supplementary file 6 — Additional file 6: Supplementary Table 2. mHFD effects on the ultrastructure of dark cells in the dorsal hippocampus CA1 stratum radiatum of PND30 offspring. [file 12974_2020_1914_MOESM6_ESM.docx]

**Supplementary Table 2. mHFD effects on the ultrastructure of dark cells in the dorsal hippocampus CA1 *stratum* *radiatum* of PND30 offspring .** #: number, CD: control diet, ER/golgi: endoplasmic reticulum and Golgi apparatus cisterna, mHFD: maternal high-fat diet, N/A: not applicable

| **Parameters** | | | **Mean ± standard error of the mean** | | | |
| --- | --- | --- | --- | --- | --- | --- |
|  |  |  | **Male** | | **Female** | |
|  |  |  | **CD** | **mHFD** | **CD** | **mHFD** |
| # Dark perivascular cells | | | 0 | 0 | 5 | 3 |
| # Dark microglia | | | 0 | 2 | 1 | 1 |
| Organelles | # Lysosomes | Primary | N/A | 1.000 ±1.000 | 1.500 ±0.619 | 1.500 ±0.645 |
|  |  | Secondary | N/A | 0.000 ±0.000 | 0.333 ±0.211 | 0.000 ±0.000 |
|  |  | Tertiary | N/A | 0.000 ±0.000 | 0.200 ±0.200 | 0.000 ±0.000 |
|  | # Lipofuscin | | N/A | 0.000 ±0.000 | 0.000 ±0.000 | 0.500 ±0.500 |
|  | # Endosome | Empty | N/A | 0.500 ±0.500 | 0.833 ±0.654 | 0.000 ±0.000 |
|  |  | Content | N/A | 1.000 ±1.000 | 0.500 ±0.342 | 0.250 ±0.250 |
|  | # Dilated ER/golgi | | N/A | 14.00 ±8.00 | 8.833 ±2.664 | 12.75 ±4.27 |
|  | # Mitochondria | Elongated | N/A | 2.500 ±2.500 | 0.167 ±0.167 | 2.500 ±1.500 |
|  |  | Total | N/A | 17.50 10.50 | 6.000 ±1.673 | 11.25 ±3.35 |
| Interactions with microenvironment | # Synaptic terminal | Pre | N/A | 18.00 ±12.00 | 4.333 ±2.431 | 4.000 ±3.674 |
|  |  | Post |  | 6.000 ±4.000 | 1.833 ±1.138 | 3.000 ±3.000 |
|  | # Myelinated axon | | N/A | 1.000 ±1.000 | 0.000 ±0.000 | 0.000 ±0.000 |
|  | # Degenerating myelin | | N/A | 1.500 ±1.500 | 0.667 ±0.333 | 0.000 ±0.000 |
|  | # Contacts with brain cells | Astrocyte | N/A | 0.000 0.000 | 0.167 0.167 | 0.000 0.000 |
|  |  | Microglia | N/A | 0.000 0.000 | 0.000 0.000 | 0.000 ±0.000 |
|  |  | Neuron | N/A | 0.000 ±0.000 | 0.000 ±0.000 | 0.000 ±0.000 |
|  |  | Oligodendrocyte | N/A | 0.000 ±0.000 | 0.000 ±0.000 | 0.000 ±0.000 |
|  |  | Blood vessel | N/A | 0.500 ±0.500 | 1.000 ±0.000 | 1.000 ±0.000 |
|  | # Extracellular space | | N/A | 1.500 ±1.500 | 0.667 ±0.667 | 0.750 ±0.750 |
|  | # Extracellular digestion | | N/A | 2.000 ±0.000 | 1.333 0.422 | 0.250 0.250 |
