# Supplementary material for: Microglial and peripheral immune priming is partially sexually dimorphic in adolescent mouse offspring exposed to maternal high-fat diet
Source: J Neuroinflammation. 2020 Sep 5;17:264. doi: 10.1186/s12974-020-01914-1 (PMC7487673; doi:10.1186/s12974-020-01914-1)
Supplement: Supplementary file 7 — Additional file 7: Supplementary Table 3. mHFD effects on the ultrastructure of dark cells in the stratum lacunosum moleculare of the dorsal hippocampus CA1 of PND30 offspring. [file 12974_2020_1914_MOESM7_ESM.docx]

**Supplementary Table 3. mHFD effects on the ultrastructure of dark cells in the *stratum lacunosum moleculare* of the dorsal hippocampus CA1 of PND30 offspring.** #: number, CD: control diet, ER/golgi: endoplasmic reticulum and Golgi apparatus cisterna, mHFD: maternal high-fat diet, N/A: not applicable.

| **Parameters** | | | **Mean ± standard error of the mean** | | | | ***F*** | ***p*** |
| --- | --- | --- | --- | --- | --- | --- | --- | --- |
|  |  |  | **Male** | | **Female** | |  |  |
|  |  |  | **CD** | **mHFD** | **CD** | **mHFD** |  |  |
| # Dark perivascular cells | | | 12 | 12 | 18 | 10 | N/A | N/A |
| # Dark microglia | | | 2 | 1 | 1 | 3 | N/A | N/A |
| Organelles | # Lysosomes | Primary | 1.357 ±0.608 | 3.154 ±0.799 | 2.158 ±0.514 | 1.846 ±0.576 | Sex*Diet: 2.846  Sex: 0.1645  Diet: 1.412 | Sex*Diet: 0.0973  Sex: 0.6866  Diet: 0.2399 |
|  |  | Secondary | 0.000 ±0.000 | 0.154 ±0.154 | 0.368 ±0.175 | 0.538 ±0.268 | Sex*Diet: 0.002048  Sex: 4.428  Diet: 0.8192 | Sex*Diet: 0.9641  Sex: **0.0399**  Diet: 0.3694 |
|  |  | Tertiary | 0.000 ±0.000 | 0.053 ±0.053 | 0.000 ±0.000 | 0.000 ±0.000 | Sex*Diet: 0.5787  Sex: 0.5787  Diet: 0.5787 | Sex*Diet: 0.4501  Sex: 0.4501  Diet: 0.4501 |
|  | # Lipofuscin | | 0.143 ±0.097 | 0.000 ±0.000 | 0.000 ±0.000 | 0.000 ±0.000 | Sex*Diet: 2.356  Sex: 2.356  Diet:2.356 | Sex*Diet: 0.1305  Sex: 0.1305  Diet: 0.1305 |
|  | # Endosome | Empty | 0.143 ±0.097 | 0.385 ±0.266 | 0.316 ±0.188 | 0.231 ±0.231 | Sex*Diet: 0.6365  Sex: 0.002171  Diet: 0.1464 | Sex*Diet: 0.4284  Sex: 0.9630  Diet: 0.7034 |
|  |  | Content | 0.714 ±0.266 | 0.769 ±0.281 | 0.737 ±0.200 | 0.846 ±0.421 | Sex*Diet: 0.008791  Sex: 0.02943  Diet: 0.08025 | Sex*Diet: 0.9256  Sex: 0.8644  Diet: 0.7780 |
|  | # Dilated ER/golgi | | 6.714 ±1.097 | 13.769 ±2.822 | 12.895 ±1.811 | 15.000 ±2.900 | Sex*Diet: 1.245  Sex: 2.791  Diet:4.264 | Sex*Diet: 0.2694  Sex: 0.1005  Diet: **0.0437** |
|  | # Mitochondria | Elongated | 0.571 ±0.202 | 1.769 ±0.568 | 1.263 ±0.285 | 1.077 ±0.415 | Sex*Diet: 3.375  Sex: 5.893×10^-7^  Diet: 1.803 | Sex*Diet: 0.0716  Sex: 0.9994  Diet: 0.1849 |
|  |  | Total | 6.000 ±1.301 | 9.000 ±2.204 | 7.789 ±1.696 | 9.538 ±1.996 | Sex*Diet: 0.1160  Sex: 0.4016  Diet: 1.671 | Sex*Diet: 0.7347  Sex: 0.5289  Diet: 0.2015 |
| Interactions with microenvironment | # Synaptic terminal | Pre | 1.286 ±0.873 | 1.769 ±1.769 | 1.105 ±1.105 | 2.692 ±1.495 | Sex*Diet: 0.1729  Sex: 0.07831  Diet: 0.6088 | Sex*Diet: 0.6791  Sex: 0.7806  Diet: 0.4386 |
|  |  | Post | 0.643 ±0.464 | 0.692 ±0.692 | 0.579 ±0.579 | 1.077 ±0.582 | Sex*Diet: 0.1414  Sex: 0.07230  Diet:0.2107 | Sex*Diet: 0.7083  Sex: 0.7890  Diet:0.6481 |
|  | # Myelinated axon | | 0.071 ±0.071 | 0.000 ±0.000 | 0.053 ±0.053 | 0.154 ±0.154 | Sex*Diet: 1.059  Sex: 0.6482  Diet: 0.03153 | Sex*Diet: 0.3079  Sex: 0.4242  Diet:0.8597 |
|  | # Degenerating myelin | | 0.214 ±0.155 | 0.077 ±0.077 | 0.421 ±0.268 | 0.000 ±0.000 | Sex*Diet: 0.5325  Sex: 0.1115  Diet: 2.063 | Sex*Diet: 0.4687  Sex: 0.7397  Diet: 0.1566 |
|  | # Contacts with brain cells | Astrocyte | 0.429 ±0.137 | 0.615 ±0.180 | 0.737 ±0.150 | 0.615 ±0.140 | Sex*Diet: 0.9762  Sex: 0.9762  Diet: 0.04388 | Sex*Diet: 0.3275  Sex: 0.3275  Diet: 0.8349 |
|  |  | Microglia | 0.000 ±0.000 | 0.077 ±0.077 | 0.053 ±0.053 | 0.000 ±0.000 | Sex*Diet: 1.776  Sex: 0.06243  Diet: 0.06243 | Sex*Diet: 0.1881  Sex: 0.8036  Diet:0.8036 |
|  |  | Neuron | 0.000 ±0.000 | 0.000 ±0.000 | 0.000 ±0.000 | 0.000 ±0.000 | N/A | N/A |
|  |  | Oligodendrocyte | 0.000 ±0.000 | 0.000 ±0.000 | 0.000 ±0.000 | 0.000 ±0.000 | N/A | N/A |
|  |  | Blood vessel | 1.071 ±0.071 | 1.077 ±0.077 | 1.158 ±0.086 | 1.154 ±0.104 | Sex*Diet: 0.002969  Sex: 0.8704  Diet: 6.816×10^-5^ | Sex*Diet: 0.9567  Sex: 0.3549  Diet: 0.9934 |
|  | # Extracellular space | | 0.071 ±0.071 | 0.000 ±0.000 | 0.105 ±0.072 | 0.000 ±0.000 | Sex*Diet: 0.08335  Sex: 0.08335  Diet: 2.273 | Sex*Diet: 0.7739  Sex: 0.7739  Diet:0.1373 |
|  | # Extracellular digestion | | 0.000 ±0.000 | 0.385 ±0.266 | 0.316 ±0.134 | 0.231 ±0.166 | Sex*Diet: 2.031  Sex: 0.2415  Diet: 0.8266 | Sex*Diet: 0.1597  Sex: 0.6251  Diet:0.3672 |
